# Effects of the Exposure of Human Non-Tumour Cells to Sera of Pancreatic Cancer Patients

**DOI:** 10.3390/biomedicines10102588

**Published:** 2022-10-15

**Authors:** Berina Sabanovic, Matteo Giulietti, Monia Cecati, Gaya Spolverato, Clara Benna, Salvatore Pucciarelli, Francesco Piva

**Affiliations:** 1 Department of Specialistic Clinical and Odontostomatological Sciences, Polytechnic University of Marche, 60131 Ancona, Italy; 2 Department of Surgical, Oncological and Gastroenterological Sciences, University of Padua, 35122 Padua, Italy

**Keywords:** pancreatic cancer, genometastasis, transformation, human serum, CNV

## Abstract

Pancreatic ductal adenocarcinoma (PDAC) has high metastatic potential. The “genometastasis” theory proposes that the blood of some cancer patients contains elements able to transform healthy cells by transferring oncogenes. Since findings on genometastasis in PDAC are still scarce, we sought supporting evidence by treating non-tumour HEK293T and hTERT-HPNE human cell lines with sera of PDAC patients. Here, we showed that HEK293T cells have undergone malignant transformation, increased the migration and invasion abilities, and acquired a partial chemoresistance, whereas hTERT-HPNE cells were almost refractory to transformation by patients’ sera. Next-generation sequencing showed that transformed HEK293T cells gained and lost several genomic regions, harbouring genes involved in many cancer-associated processes. Our results support the genometastasis theory, but further studies are needed for the identification of the circulating transforming elements. Such elements could also be useful biomarkers in liquid biopsy assays.

## 1. Introduction

Pancreatic ductal adenocarcinoma (PDAC) represents the most common type of pancreatic neoplasm, with a median survival of six months and a five-year survival for all stages of only 5%. Such a low survival rate is explained by its high rate of metastasis formation. Moreover, PDAC is diagnosed in the vast majority of patients in this advanced stage, who are therefore non-eligible for surgery [1,2].

Paget’s classical “seed-and-soil” theory of metastasis is not sufficient to fully describe progression of many tumours, including PDAC. Indeed, some metastatic patterns can be explained by the anatomical-mechanical hypothesis claiming that they are determined by the anatomy of vascular and lymphatic drainage at primary tumour site, and the fact that the circulating cancer cells’ arrest mainly occurs at the adjacent organ. Both hypotheses are accepted, and their validity depends on the type of tumour [3,4]. However, the dissemination of cancer cells through circulation may not be the unique principle for metastasis formation, since some phases of the metastatic process are very inefficient. Indeed, only 0.001% of circulating cancer cells can survive in the circulation and thus lead to metastasis [5].

The hypothesis of “genometastasis” represents a further model explaining metastasis. It is based on the horizontal transfer of genetical material (DNA, RNA, miRNAs, retrotransposon elements, mutated and amplified oncogenes) carried in circulation to cells in distant organs. These genetic elements in the target cells activate and promote mitogenic signalling pathways, causing the malignant transformation [5]. Circulating DNA and RNA molecules are found in several forms of molecular complexes, linked to serum proteins, or are loaded into extracellular vesicles. These molecules are passively released by apoptotic and necrotic cells, and actively secreted by almost all living cells [3]. Recently, it was experimentally demonstrated that cell-free cancer-derived DNA can transform non-tumour cells in vitro upon treatment with colorectal cancer patient plasma or by co-culturing tumour and non-tumour cells [6,7]. Abdouh et al. suggested that mutation/inactivation of tumour suppressor genes in distant recipient cells could cause the aberrant expression of membrane proteins, permitting the genome integration of circulating oncogenic factors [8].

Recently, there have been emerging studies focusing on assessing the validity of genometastasis hypothesis in colon cancer [9] and breast cancer [10,11]. The studies regarding the validation of genometastasis theory in PDAC are few. For example, Costa-Silva et al. suggested that PDAC-derived exosomal factors prime the liver cells for the metastasis development and therefore might be used as a prognostic marker [12]. Stefanius et al. found that exosomes isolated from pancreatic cancer cells act as initiators of malignant cell transformation, but in order to obtain a fully transformed state, the promoter activity of mutagen compounds is necessary [13]. There are many findings of cancer patient sera and/or cancer-derived extracellular vesicles-mediated transfer of malignant characteristics to primed cells causing their transformation [14]. The term “primed cells” refers to cells with mutated oncogenes and/or oncosuppressors, which has been shown to be a necessary but not sufficient condition for the transformation induced by cancer patient sera. For example, BRCA1 knockout fibroblasts [15,16] and PTEN deleted MCF10A cells [17] were successfully transformed by cancer patient sera. On the other hand, non-tumour cells have been shown to be refractory to the transformation potential of serum of cancer patients [5].

The confirmation of genometastasis theory would allow the identification of patients who could benefit from the surgery and those who, having already developed the micrometastasis, are not eligible for pancreatomy. The identification of molecules, present in the patients’ sera, driving the malignant transformation would allow the development of novel liquid biopsy assays. Additionally, it would be possible to identify the drugs inhibiting such molecules and therefore to develop better and personalized therapeutic approaches.

In this work, we aimed to obtain more results supporting the genometastasis theory in the setting of PDAC, exposing two primed cell lines, hTERT-HPNE and HEK293T, to PDAC patients’ sera. In particular, we analysed the transformation potential of patients’ sera, and the changes in protein expression, migration, invasion, and chemoresistance induced by serum treatments. Next-generation sequencing analysis allowed the identification of gained and lost genomic traits, which harbour cancer-associated genes that may explain the observed phenotypic changes.

## 2. Materials and Methods

### 2.1. Cell Lines and Culture Conditions

As target cells for assessment of malignant transformation, we used hTERT-HPNE E6/E7/K-RasG12D (CRL-4038, ATCC, Manassas, VA, USA) and HEK293T XPack CMV-XP-GFP-EF1Puro (XPAK530CL-1, System Biosciences, Palo Alto, CA, USA). The hTERT-HPNE cell line represents the intermediary stage during acinar-to-ductal metaplasia in pancreas. They have an undifferentiated phenotype and active Notch signalling pathway. The HEK293T cell line is a highly transfectable derivative of human embryonic kidney 293 cells and contains the SV40 T-antigen. Both hTERT-HPNE and HEK293T cell lines were maintained in Dulbecco’s Modified Eagle Medium (ECM0749L, EuroClone, Milan, IT) supplemented with 10% of either PDAC patients’ serum or healthy donor serum (without FBS), 1 mM L-glutamine (BE17, 605E, Lonza, Verviers, BE), 1 mM penicillin-streptomycin (ECB3001D, EuroClone), and 1% of MEM non-essential amino acids for HEK293T cell line.

### 2.2. Serum Treatments

We obtained pooled PDAC patients’ serum from Tissue Biobank of the 1st Surgical Clinic, University Hospital of Padova, Italy. The University-Hospital Ethics Committee of Padua approved the study protocol (Prot. No. P 480/2002). The patients’ sera were drawn before the chemotherapy, and therefore they were free from any drugs. We have pooled sera from 25 patients with a medium age of 69 years, both male and female. The pooled control serum was obtained from 25 healthy volunteers with a medium age of 60 years. Both healthy donor and PDAC patients’ sera were filtered with 0.22 um sterile filters. The recipient cell cultures were supplemented with 10% of PDAC or healthy donor serum instead of FBS, and the medium was changed every 2 days for 3 weeks, as previously described [17].

### 2.3. Soft Agar Colony Formation Assay

At the end of the serum treatments, we assessed cell transformation in vitro using soft agar colony formation assay. The test was done in triplicate for each condition. This assay is based on the anchorage-independent growth of tumour cells whereas non-tumour cells are unable to proliferate. Sterile solutions of agar at concentrations of 1% and 0.6% were prepared in deionized water. Cell culture medium was prepared at 2X concentration and sterilized. The 6-well dishes were used for the assay. The bottom layer of plate was made of 1% agar and 2X cell culture medium in the ratio 1:1. Upon solidification, the upper layer was prepared of 0.6% agar and cell suspension in medium in a ratio 1:1. The agar was allowed to solidify at room temperature in cell culture hood for 30 min before placing it into an incubator at 37 °C and 5% CO_2_. The 6-well plate was incubated for 21 days, with the addition of 100 μL of fresh medium twice a week to prevent desiccation. Colonies were visualized and photographed under the Eclipse Ti2E microscope (Nikon, Tokyo, Japan) and we counted only those larger than 50 μm.

### 2.4. Wound Healing Migration Assay

Upon healthy or PDAC serum exposure, migration assay was performed, as by Cecati et al. [18]. Briefly, cells were plated in 24-well plates and allowed to attach for 24 h. When cells reached the confluence, they were starved in DMEM without FBS for 24 h before running the assay. The scratch was applied with a sterile 1000 μL pipette tip. Then, the wells were washed three times with PBS buffer to remove the detached cells and cell debris, and fresh DMEM without FBS was added. The assay for each condition was done in triplicate. The images were taken at different time points until the gap was closed (at 0 h, 6 h, 24 h, and 48 h), by using the Eclipse Ti2E microscope (Nikon, Tokyo, Japan).

### 2.5. Invasion Assay

Invasion assays were carried out using 8 μm pore Costar transwells (#3428, Corning, Cambridge, MA, USA), according to manufacturer protocol. Inserts were coated with 50 μL (0.50 mg/mL) Matrigel (#354234, Corning). Pre-treated cells were plated in the upper chamber in FBS free DMEM, whereas DMEM with 10% FBS (as the chemo-attractant) was added to the bottom chamber. Cells were allowed to invade for 48 h, and those that had not crossed the membrane were removed by scrubbing with a cotton swab. Then, the lower surface of the inserts was fixed in 100% methanol for 10 min at room temperature and stained with 0.3% crystal violet. Cells on the stained membrane were counted under Eclipse Ti2E microscope (Nikon). Each experiment was performed in triplicate and the data were presented as mean ± SD.

### 2.6. Western Blot

Cells were lysed in RIPA buffer containing protease and phosphatase inhibitors. Proteins were run on 8% or 12% polyacrylamide gel and transferred to a nitrocellulose membrane (#10600006, GE Helathcare Life science, DE). Membranes were blocked in TBS buffer (20 mM TRIS, 150 mM NaCl, pH 7.4) containing 5% Bovine Serum Albumin and exposed to rabbit-anti-mTOR (#2983, Cell Signalling, Danvers, MA, USA), rabbit-anti-phospho-mTOR (#5536, Cell Signalling), rabbit-anti-FN1 (#26836, Cell Signalling), rabbit-anti-phospho-Stat3 (#9145, Cell Signalling), rabbit-anti-Stat3 (#4904, Cell Signalling), rabbit-anti-Vimentin (#3932, Cell Signalling), rabbit-anti-GAPDH (#2118, Cell Signalling), rabbit-anti-PD-L1 (#13684, Cell Signalling), rabbit-anti-PCNA (#13110, Cell Signalling), rabbit-anti-N-Cadherin (#4061, Cell Signalling), and rabbit-anti-E-Cadherin (#3195, Cell Signalling) overnight at 4 °C. Membranes were washed in TBST (TBS-0.05% Tween-20) and incubated with either anti-rabbit HRP-conjugated secondary antibody for 1 h at room temperature. After several washes in TBST, the blots were developed using SuperSignal West Pico PLUS Chemiluminescent Substrate (#34580, Thermo Fisher Scientific, Waltham, MA, USA).

### 2.7. Chemoresistance

To assess whether the cell lines treated with PDAC serum have acquired chemoresistance, we selected anticancer drugs. In particular, we assessed cellular viability upon treatments with Gemcitabine (#G6423, Sigma, St. Louis, MO, USA), Doxorubicin (CAS number 23214-92-8), 5-fluorouracil (#F6627, Sigma), and Paclitaxel (CAS number 33069-62-4). Treated and control cells were seeded 20,000 per well and incubated with drugs at their IC50 concentration for 72 h. Subsequently their vitality was assessed by MTT assay. The MTT assay is based on the reduction of a yellow tetrazolium salt (#A2231,0001, Applichem GmbH, Darmstadt, Germany) to purple formazan crystals by metabolically active cells. The insoluble formazan crystals were dissolved using DMSO (#EMR385100, EuroClone), and the resulting coloured solution was quantified by measuring absorbance at 570 nm using a multi-well spectrophotometer. All tests were done in triplicate for each condition. Finally, we compared the cell viability between treated and control cell lines.

### 2.8. Whole Genome Sequencing (WGS) and Data Analysis

To minimize the influence of possible genomic instability, we split HEK293T cells into two plates (control and treated) starting from the same flask, and we cultured the in parallel and analysed at the same number of passages. The genomic DNA was isolated from HEK293T cells after exposure to PDAC patients’ sera and healthy donor sera using Exgene^TM^ Clinic SV mini extraction kit (Cat. No. 108–101, GeneAll biotechnology Ltd., Seoul, Korea). DNA was treated with RNase, eluted in 40 µL of deionized water, and run on 1% agarose gel for integrity evaluation. The total sample quantity was determined on Qubit fluorometer (Cat. No. Q33226, Invitrogen, Waltham, MA, USA). The WGS was carried out on the NovaSeq^TM^ 6000 platform (Cat. No. 20012850, Illumina, San Diego, CA, USA) in a Pair-End 2 × 150 bp setup.

After quality control by FASTQC tool, the files containing the WGS paired-end reads were aligned against the human genome hg38 by using BWA-MEM tool (ver. 0.7.17) [19], with default parameters. The produced BAM files (treated and the matched normal control samples) were submitted to the well-known copy number caller Control-FREEC (ver. 11.0) [20], using default parameters. This tool is based on read-depth alignment and automatically computes, normalizes, and segments copy number profiles for the detection of CNVs (copy number variations), and calculates their significance (by Wilcoxon and Kolmogorov–Smirnov tests).

### 2.9. Statistical Analyses

For colony formation, migration, invasion, and MTT assays, significant differences between the treated and untreated cells were determined using the t-test. P values of less than 0.05 were considered statistically significant. All statistical analyses for the abovementioned assays were performed by using the Stat6 Software for Windows (Stat6 Software, San Diego, CA, USA). The Wilcoxon test and Kolmogorov–Smirnov test were used for analysis of WGS-CNV data.

## 3. Results

### 3.1. Serum Treatments and In Vitro Transformation Validation

**Figure 1:**
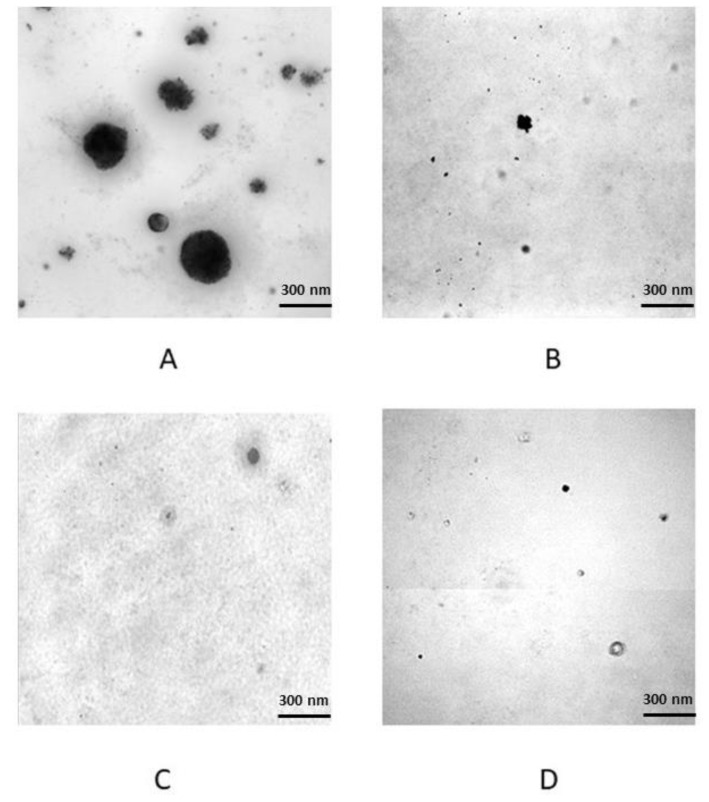
The representative colonies in soft agar colony formation assay: (**A**) HEK293T + PDAC serum; (**B**) hTERT-HPNE + PDAC serum; (**C**) HEK293T + healthy donor serum; (**D**) hTERT-HPNE + healthy donor serum. Note that the horizontal lines in (**B**, **D**) figures were caused by rolling shutter modality of the microscope camera (Hamamatsu ORCA-Flash4.0 LT).

In order to evaluate the transforming potential of PDAC patient serum, we treated two non-tumour cell lines, hTERT-HPNE and HEK293T, with pooled sera from PDAC patients and from healthy subjects. The soft agar colony formation assay was done in triplicate for each condition, and Figure 1 shows some representative images of the colonies of hTERT-HPNE and HEK293T cells formed in soft agar upon PDAC serum exposure and their respective controls. The average colony number formed by HEK293T cell line treated with PDAC patients’ sera was 54 ± 0.81 with a medium colony size of 136 ± 69.9 µm, whereas the same cell line treated with healthy sera did not form colonies larger than 50 µm. Similarly, for the healthy sera treatment of hTERT-HPNE line, colonies larger than 50 µm were not observed. Moreover, hTERT-HPNE cells treated with PDAC patients’ sera formed an average of 6 ± 0.47 colonies with a medium size of 64 ± 18.5 µm.

Overall, these results suggest that PDAC patients’ sera has the potential to transform non-tumour cell lines, and that HEK293T cells seem to be more prone to malignant transformation than hTERT-HPNE cells.

### 3.2. Cell Migration and Invasion Assay

**Figure 2:**
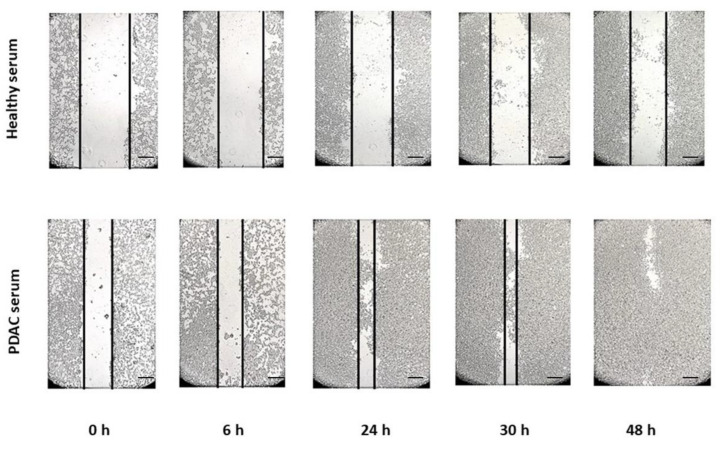
The representative wound healing migration assay for HEK293T cell line treated with healthy or PDAC sera. Scale bar = 400 μm.

**Figure 3:**
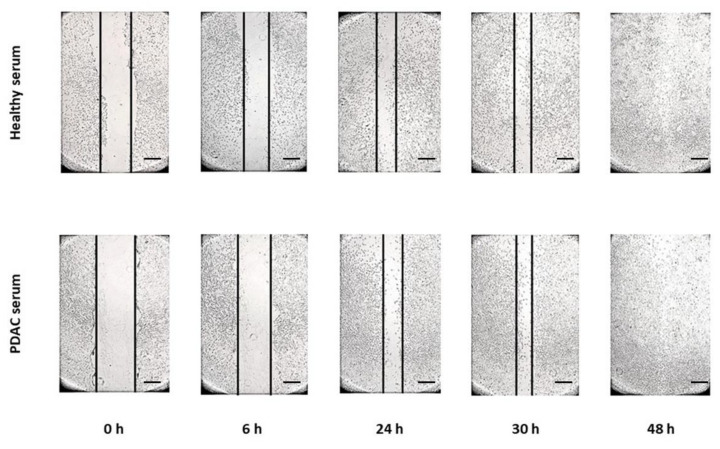
The representative wound healing migration assay for hTERT-HPNE cell line treated with healthy or PDAC sera. Scale bar = 400 μm.

The wound healing assay was used to reveal changes in migration velocity and cell–cell interaction. Figure 2 and Figure 3 show some representative images (all plates were done in triplicate) of the wound healing migration assay for hTERT-HPNE and HEK293T cell lines until the gap closure.

**Figure 4:**
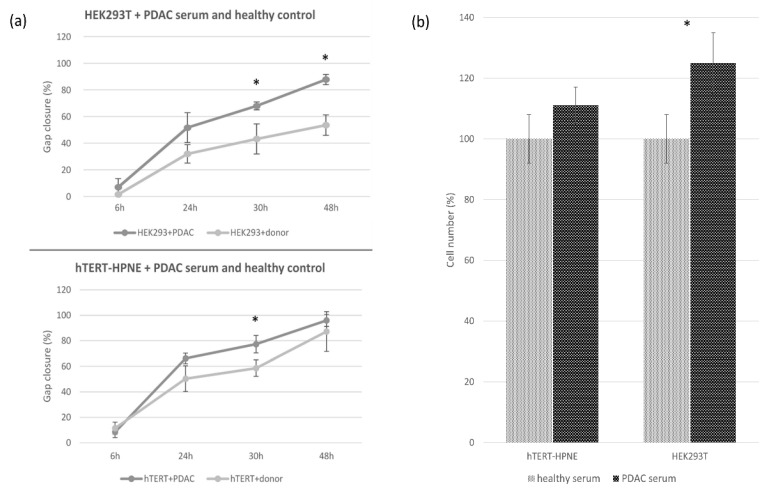
Migration (**a**) and invasion (**b**) assays. Graphical representation of mean values with standard deviation for HEK293T and hTERT-HPNE cell lines treated with PDAC serum compared to the healthy serum. Significant differences (*p* < 0.05) are marked with an asterisk.

The graphs in Figure 4 summarize the results of migration and invasion assay for both cell lines and conditions. The HEK293T cell line treated with PDAC serum completely closed the gap after 48 h, whereas the control group at the same time point closed the gap only at about 55% (*p* < 0.05). The migration of hTERT-HPNE cells treated with PDAC serum was not much different from the control group, in particular, after 48 h treated cells have closed the 95% of the gap and the control cells at 87% (Figure 4a). Similarly, the invasion’s abilities resulted in being slightly enhanced in the hTERT-HPNE cell line (+11%, not significant) and strongly increased in the HEK293T cells (+25%, *p* < 0.05) after treatment with serum of PDAC patients (Figure 4b). Taken together, these results indicate that the treatment with PDAC serum stimulated the aggressive behaviour of HEK293T cells, whereas hTERT-HPNE cells did not show this effect.

### 3.3. Protein Expression in Western Blot

**Figure 5:**
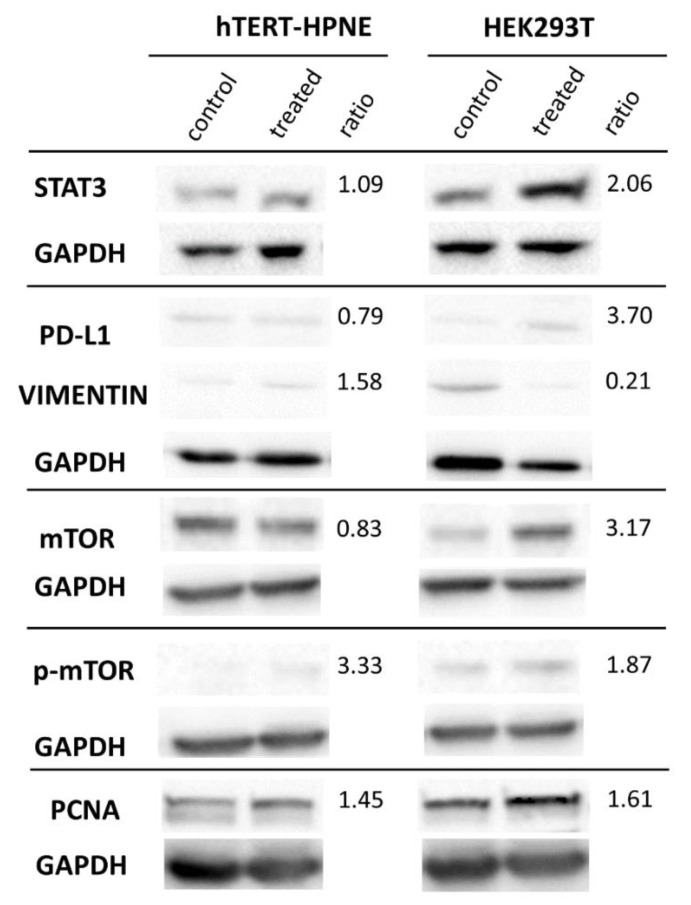
The representative western blot for vimentin, mTOR, p-mTOR, PCNA, PD-L1, and STAT3 each with the corresponding GAPDH protein used for data normalization. The lanes correspond to the following samples: (control) hTERT-HPNE or HEK293 + healthy serum; (treated) hTERT-HPNE or HEK293 + PDAC serum; (ratio) Expression ratio between PDAC and healthy serum treatments in hTERT-HPNE and HEK293T cell lines.

Total proteins were extracted from both PDAC serum-treated cell lines and respective controls in order to analyse the expression of typically up- and down-regulated proteins in PDAC tumour cells. These chosen proteins play key roles in some signalling pathways, in epithelial-to-mesenchymal transition (EMT) and enable immuno-escape of tumour cells. The representative images of membranes for antibodies against expressed proteins along with expression ratio between PDAC serum-treated cells and controls are presented in the Figure 5. Membranes for the proteins which resulted in not being expressed are presented in the Supplementary Figure S1.

The HEK293T cell line showed an up-regulation of proteins responsible for increased growth, proliferation (PCNA, STAT3, mTOR) after PDAC serum treatment. In particular, expression of STAT3 is 2-fold higher, and the expression of mTOR and its active phosphorylated form, p-mTOR, were 3-fold and 2-fold higher, respectively. In addition, p-STAT3 is absent in both conditions. The expression of PD-L1, responsible for immuno-escape of tumour cells, is 3.7-fold higher. The only down-regulated protein in HEK293T cell line is vimentin (−4.76-fold).

Interestingly, in the hTERT-HPNE cell line, we observed the opposite situation. In particular, the vimentin expression was slightly up-regulated (1.58-fold), the mTOR expression was slightly down-regulated, and PCNA, PD-L1, and STAT3 did not show notable (>1.5 or <0.66) expression changes in this cell line.

The common feature for both cell lines was a complete absence of fibronectin, E-cadherin, and N-cadherin. In order to validate antibodies’ functionality, we assessed fibronectin, E-cadherin, and N-cadherin in HEK293T, hTERT-HPNE, and other PDAC cell lines not treated with human serum (Supplementary Figure S2).

### 3.4. Chemoresistance

Since all previous results indicated that the HEK293T cell line has gained more changes in terms of protein expression, migration, invasion, and malignant transformation, we exposed it to common chemotherapeutics in order to validate its response. At the end of the incubation time, we assessed the IC50 based on cell viability using the MTT assay in triplicate for each drug condition. The results are summarized in Table 1.

The transformed HEK293T cell line has developed resistance to paclitaxel. Indeed, upon exposure to 100 nM paclitaxel, the control HEK293T cells had an average viability of about 50%, whereas the PDAC serum-treated cells had a 91% of viability. Gemcitabine, doxorubicin, and 5-FU caused an opposite effect, reducing the viability of PDAC serum-treated cells.

### 3.5. Genomic Imbalance in PDAC Serum-Treated Cells and Literature Analysis

**Figure 6:**
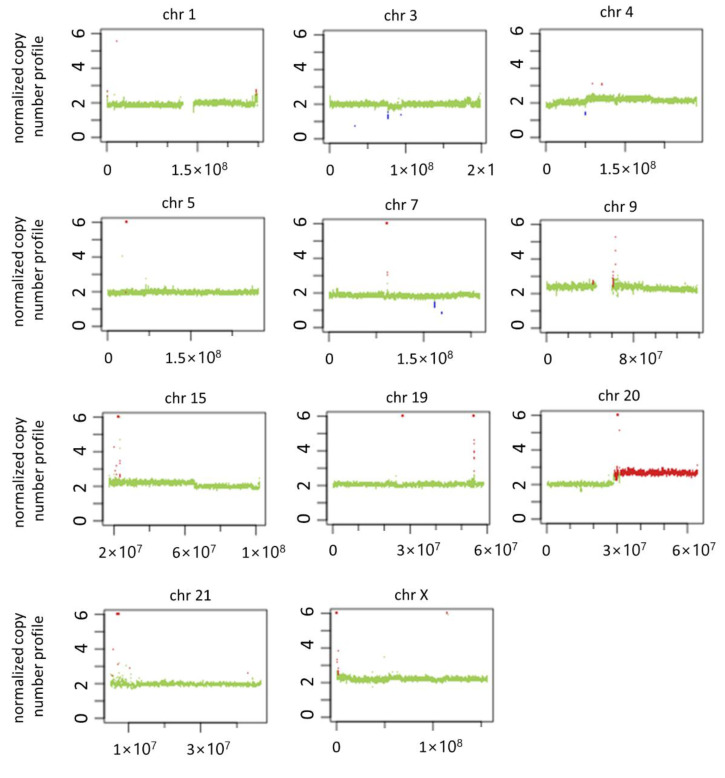
These graphs summarize the results of the chromosomal gain or loss events of HEK293T cells, treated with plasma from patients, compared to cells treated with plasma from healthy subjects. An image is shown for each chromosome where we found statistically significant results (other chromosomes in Supplementary Figure S3). The chromosomal position is shown on the abscissa axis. The number of copies of each specific chromosomal segment is shown in the ordinate axis. In the absence of CNV events, values equal to 2 (diploidy) are expected. Unfortunately, the size of this image does not allow for a good appreciation of the CNV events, so we have included the high-magnification image (Supplementary Figure S4), and more details are shown in Table 2.

After we established that HEK293T cells were transformed upon PDAC serum treatments, for the first time, we assessed the gene copy number variations (CNVs) using NGS approach and compared them to untreated cells. The obtained paired-end sequences from genomic DNA were aligned to human genome hg38 and submitted to the copy number calling tool Control-FREEC. We have identified both gains and losses in various chromosomes (Figure 6 and Table 2).

In particular, losses have been detected located on chromosomes 3p, 4, and 7q, and, interestingly, the unique genes with roles in tumour-related process in these regions (FBXL2, ROBO2) are tumour suppressors (Supplementary Table S1).

We have observed major gains in regions of chromosomes 19q and 20q, and some modest changes in chromosomes 1, 4p, 5p, and 9. Notably, these amplified regions harbour 70 genes involved in tumour-related processes in PDAC and/or other solid tumours. The majority (49 genes) have pro-tumour effects (i.e., higher expression in tumours, associated with lower survival, able to induce cell proliferation, migration, invasion, EMT, metastasis, stemness, chemoresistance or inhibition of apoptosis), 15 genes act as tumour suppressors, and six genes have still undefined or both pro-tumour and anti-tumour activities (Supplementary Table S1). In particular, most of the amplified genes are involved in proliferation and tumour progression in PDAC, such as AURKA, HRH3, MIR646, MRGBP, PCK1, PMEPA1, SLCO4A1-AS1, SOX18, and TNFRSF6B. Some of the amplified genes are frequently overexpressed in PDAC and are related to a poor prognosis (BIRC7, EEF1A2, NTSR1, RAB22A, TNFRSF6B). Other amplified genes are involved in migration, invasiveness, and EMT in PDAC, such as BIRC7, EEF1A2, LAMA5, MIR646, MRGBP, PCK1, PMEPA1, PTK6, SLCO4A1-AS1, TNFRSF6B, and ZNF217. Furthermore, the amplified genes BIRC7, EEF1A2, MIR646, NTSR1, and TNFRSF6B play a role in PDAC metastasis, while AURKA and SLCO4A1-AS1 are involved in apoptosis. Another important feature of analysed genome was amplification of genes responsible for drug metabolism and chemoresistance in PDAC, such as AURKA, CDH4, TFAP2C, PMEPA1, and PTK6. Among amplified genes with anti-tumour roles in PDAC, we identified four microRNA genes (MIR1-1, MIR124-3, MIR133A2, and MIR296). They act as tumour suppressor genes, are downregulated in PDAC and associated with prognosis, and they can suppress cell proliferation, invasion, migration, and EMT, or enhance drug sensitivity of pancreatic cancer cells (see Supplementary Table S1 for details).

Some of the CNV chromosomal regions that we identified do not contain known genes, but it cannot be excluded that these regions are, in any case, able to contribute to general genomic instability in the transformed cells.

## 4. Discussion

PDAC is one of the most lethal cancer types and the majority of patients are diagnosed at the advanced stages, which narrows down the choice of therapeutic approaches. Furthermore, PDAC has a very high metastatic potential, and metastases have been observed even in patients who have undergone a complete pancreatic resection [21]. All of this highlights the urgency to understand better the metastatic development of PDAC and to identify the targets of malignant transformation in distant cells.

In order to contribute to the understanding of the genometastasis theory in PDAC, we have tested the transformation ability of non-metastatic PDAC patients’ sera on primed cell lines hTERT-HPNE and HEK293T. Here, we have demonstrated, in vitro, that HEK293T cell line treated with PDAC patients’ serum has most likely been transformed, whereas the treated hTERT-HPNE cell line formed only a few colonies in soft agar colony formation assay. Both human cell lines are primed, with the hTERT-HPNE representing an intermediary-stage in PDAC pathogenesis with K-Ras (G12D) mutation, inactivated p53 and Rb tumour suppressor genes [18], and HEK293T cell line derived from HEK293 line upon insertion of SV40 T antigen, which inhibits p53 and Rb activity [22]. The more efficient transformation observed in HEK293T could be explained by its high susceptibility to transfection. Indeed, this cell line is widely used in many studies for its well-known efficiency and high reproducibility in exogenous protein production. In a similar study published by Abdouh et al., HEK293 cell line was exposed to cancer patients’ sera (including one case of PDAC) and the malignant transformation was confirmed, whereas other normal human cell lines did not undergo malignant transformation [8,16]. Literature data regarding hTERT-HPNE cell line are not available, since our study is the first so far reported where this cell line has been exposed to the PDAC patients’ sera to assess the transformation.

Here, by NGS analysis, we identified genomic changes (CNVs) in the HEK293T cell line after PDAC serum treatments. In particular, major gains have been detected in chromosomes 19q and 20q, and some modest changes in chromosomes 1, 3, 4, 5, 7, and 9. In PDAC, the genetic gains on chromosomes 19q and 20q and the loss on chromosomes 3p and 4 have already been described [23]. Moreover, the main amplified region (20q) is known to be duplicated in many cancer types, including PDAC, which occurs at the early transformation stages [24]. More recently, it was shown that 20q gain was harboured in more than 80% of tested PDAC patients and in about 60% of intraductal papillary mucinous neoplasm (IPMN), a precursor lesion of pancreatic cancer [25]. These results suggest that 20q amplification may occur early in the tumour development. In addition, within the 20q arm, the gene CTSZ was the most frequently amplified gene in pancreatic cancer [26]. Additionally, in our study, we identified this amplified gene, which is a tumorigenic protease able to promote tumour cell proliferation by interacting with integrins [27] (Supplementary Table S1). Further possible events that favoured transformation are the gain of the oncogenes GNAS (a PDAC driver gene), LINC00659 (an oncogene in colorectal cancer), and ZNF217 (an oncogene in many solid tumours, including PDAC). The overexpression of many other amplified genes (e.g., AURKA, HRH3, MIR646, MRGBP, PCK1, PMEPA1, SLCO4A1-AS1, SOX18, TNFRSF6B) is associated with PDAC cell proliferation and tumour growth. In addition, two tumour suppressor genes have been lost in the transformed HEK293T, namely FBXL2, able to induce cell cycle arrest, and ROBO2, which inhibits PDAC cell proliferation, migration, and invasion (see Supplementary Table S1 for details).

Although HEK293T’s high genomic instability could be expected due to the inhibition of p53 and Rb, there is reassuring evidence regarding the validity of the NGS analysis. Yao-Cheng Lin et al. compared three HEK293T cell lines which differed in the number of passages or laboratory origin. These lines clustered very tightly together for SNP content, whole-genome CNV, and gene copy number. Therefore, the authors concluded that these different HEK293T strains were highly similar at a genomic level [22]. Moreover, analogous conclusions have been pointed out for other cell lines, of which as many as 27 different strains have been analysed [28].

We have also assessed if our cell lines treated with PDAC patients’ sera have acquired higher migratory potential than cells treated with sera from healthy donors. While hTERT-HPNE cell line did not show a gain of migratory potential, HEK293T showed higher cell migration. This ability is important for tumour invasiveness, angiogenesis, and metastasis development [29,30]. Therefore, our results indicate that HEK293T cells treated with PDAC serum have acquired a more aggressive phenotype. Interestingly, STAT3 has been shown to play a role in promoting cell migration [31]. Our data show that the expression of STAT3 in HEK293T is strongly increased, whereas it is unaltered in hTERT-HPNE, consistent with the observed results of wound healing migration assay. We have observed an increase of STAT3 expression in the HEK293T cell line, but its active phosphorylated form was absent both in PDAC and healthy sera-treated cells. Although STAT3 is known to be constitutively activated in PDAC by phosphorylation of Tyr705 [32], the lack of p-STAT3 expression in untreated HEK293 cells has been already reported [33,34]. However, the unphosphorylated STAT3 can play a role in the transcriptional regulation of many cell-cycle genes through a different mechanism [35].

We also observed an up-regulation of mTOR and its phosphorylated form in PDAC serum-treated HEK293T cells. Notably, the hTERT-HPNE line showed an opposite behaviour, i.e., although p-mTOR also increased in hTERT-HPNE, the overall mTOR expression decreased after PDAC serum treatments, in contrast to HEK293. The normal mTOR signalling pathway is altered in many cancers and its activity is increased in PDAC. Moreover, since mTOR not only improves cell proliferation, growth, and survival but also drives the tumour cell motility and invasiveness [36,37], the observed expression alterations may explain the higher migration levels of treated HEK293T than hTERT-HPNE cells. The enhanced migratory abilities of treated HEK293T can also be due to the amplification of genes already known to promote migration and invasion in PDAC, such as EEF1A2, LAMA5, MRGBP, PCK1, PMEPA1, PTK6, and RUFY3 [38,39,40].

Regarding markers of epithelial-to-mesenchymal transition (EMT), we identified only a differential expression of vimentin, a mesenchymal marker. In particular, we have observed its strong reduction in HEK293T cell line and a slight increase in hTERT-HPNE cell line upon PDAC serum treatment. Since vimentin up-regulation is correlated with PDAC development and metastatic behaviour [41,42,43], PDAC serum treatments could make hTERT-HPNE cell more aggressive than HEK293T. However, other mesenchymal markers (N-cadherin and fibronectin) and the epithelial marker E-cadherin were not expressed in any conditions in both cell lines. Previously, it was reported that E-cadherin and fibronectin were not expressed in HEK293 cells [44,45] and both E- and N-cadherin were not expressed in a pancreatic cancer cell line [46]. Taken together, these results may indicate an incomplete EMT switch. However, some amplified genes in treated HEK293T (e.g., BMP7, MRGBP, CDH12) are associated with EMT in PDAC and other cancer types [47,48,49,50]. 

According to literature [51], we also observed a co-expression between STAT3 and PD-L1 expression in both cell lines. In particular, we showed an overexpression of both proteins in HEK293T, and their decrease in the hTERT-HPNE cell line after PDAC serum treatments. PD-L1 is up-regulated in numerous human cancers, including PDAC, causing immuno-escape of cancer [52]. The PD-L1 overexpression in HEK293T line may indicate that the treatment with PDAC serum made these cells able to avoid the immune response mediated by T-cells.

Interestingly, we observed the development of paclitaxel chemoresistance in HEK293T cells after PDAC serum treatments. The paclitaxel resistance could be due to the observed mTOR and p-mTOR increase. Indeed, it has been reported that inactivation of mTOR increased the sensitivity of cells to paclitaxel in cervical cancer cells [53]. Furthermore, three genes that we found to be amplified in serum-treated HEK293T cells (i.e., AURKA, RAE1, and ZFP64) are known to increase the resistance of pancreatic, colorectal, and gastric cancer cells to paclitaxel [54,55,56].

On the contrary, treated HEK293T resulted in being more sensitive to the other tested drugs commonly used in treatments for PDAC and other cancers (gemcitabine, 5-fluorouracil, and doxorubicin). The observed higher sensitivity of treated HEK293T may be due to the mechanism of action of these drugs, which is very efficient on fast proliferating cells. Indeed, HEK293T cells have a higher proliferation upon treatments with cancer patient serum, as previously described by Abdouh et al. [8]. This high proliferation of serum-treated HEK293T is also supported by the observed up-regulation of PCNA, mTOR, and STAT3 expression, known to be involved in cell growth and proliferation. Additionally, some amplified genes detected by NGS-CNV analysis in PDAC sera-treated HEK293T cells have been previously associated with increased sensitivity to 5-FU and gemcitabine. In particular, when delivered by exosomes, the miR-124 enhanced 5-FU sensitivity of pancreatic cancer cells [57]. Moreover, overexpression of CDH4, PTK6, and TFAP2C re-sensitizes pancreatic cancer cells to gemcitabine [58,59,60].

## 5. Conclusions

In this study we added results in the support of genometastasis theory in the setting of PDAC. We have demonstrated in vitro that the HEK293T non-tumour cell line exposed to PDAC patients’ sera has acquired a transformed phenotype, gained aggressiveness, and gained resistance to paclitaxel. NGS analysis showed the gain of genes already known to be involved in proliferation, migration, invasion, EMT, metastasis, and chemoresistance. However, the transforming molecules released by PDAC and carried by plasma are still unknown, although there are early indications that they are transferred through extracellular vesicles. For these reasons, it will be interesting to characterize the nucleic acid and protein content of these vesicles. Once these molecules are identified, they could be therapeutic targets and prognostic biomarkers indicating the presence of micro-metastases and therefore an exclusion criterion for surgery.

## Figures and Tables

**Table 1:** The MTT assay results for cell lines treated with chemotherapeutics expressed as average percentage of cell viability. HEK293T (control): previously treated with serum of healthy volunteers. HEK293T (treated): previously treated with serum of PDAC patients.

Drugs	HEK293T (Control)	HEK293T (Treated)	*p*-Value
Gemcitabine 13 uM	42%	16%	0.009
Doxorubicin 100 nM	53%	30%	0.001
Paclitaxel 100 nM	51%	91%	0.001
5-FU 5 uM	46%	17%	0.001

**Table 2:** WGS-CNV analysis. List of the genes present in copy number variation in the genome of HEK293T cell line treated with PDAC patients’ sera.

Coordinates (Chromosome: Start–End)	Status (Copy Number)	*p*-Value (Wilcoxon Rank Sum; Kolmogorov–Smirnov)	Genes
1: 246,640,000–246,749,999	gain (3)	4.34 × 10^−2^; 2.55 × 10^−1^	CNST, SCCPDH
3: 33,290,000–33,379,999	loss (1)	1.11 × 10^−4^; 9.40 × 10^−5^	FBXL2
3: 76,610,000–76,719,999	loss (1)	6.26 × 10^−6^; 2.23 × 10^−6^	ROBO2
3: 93,430,000–93,519,999	loss (1)	1.40 × 10^−4^; 1.09 × 10^−4^	none
4: 49,660,000–49,759,999	loss (1)	2.91 × 10^−5^; 1.55 × 10^−5^	none
4: 70,690,000–70,779,999	gain (3)	1.37 × 10^−3^; 5.15 × 10^−4^	RUFY3, UTP3
5: 22,290,000–22,379,999	gain (6)	2.76 × 10^−2^; 4.79 × 10^−3^	CDH12
7: 111,400,000–111,579,999	loss (1)	1.46 × 10^−10^; 2.54 × 10^−12^	IMMP2L
7: 119,650,000–119,739,999	loss (1)	1.15 × 10^−4^; 9.62 × 10^−5^	LINC02476
9: 61,000,000–61,509,999	gain (3)	2.23 × 10^−6^; 4.67 × 10^−6^	SPATA31A7, FAM74A4, CNTNAP3C
15: 23,180,000–23,239,999	gain (3)	3.63 × 10^−2^; 6.56 × 10^−2^	none
19: 54,730,000–54,779,999	gain (4)	4.25 × 10^−4^; 3.21 × 10^−4^	KIR3DL3, KIR2DL1
19: 54,740,000–54,789,999	gain (38)	3.71 × 10^−4^; 3.21 × 10^−4^	KIR3DL3, KIR2DL1
19: 54,750,000–54,839,999	gain (4)	2.93 × 10^−6^; 4.53 × 10^−6^	KIR3DL4, KIR3DL3, KIR2DL1
19: 54,800,000–54,869,999	gain (8)	1.77 × 10^−3^; 6.15 × 10^−3^	KIR2DL4, KIR3DL1, KIR2DS4, KIR3DL2
20: 29,120,000–30,079,999	gain (3)	8.02 × 10^−22^; 0.00	FAM242B, FRG1EP, FRG2EP
20: 30,040,000–30,089,999	gain (114)	2.14 × 10^−3^; 3.09 × 10^−3^	none
20: 30,050,000–30,359,999	gain (3)	1.01 × 10^−2^; 2.62 × 10^−2^	FAM242A, LINC01597
20: 51,700,000–52,099,999	gain (3)	6.01 × 10^−20^; 0.00	ATP9A, SALL4, LINC01429, ZFP64
20: 52,100,000–52,989,999	gain (3)	1.01 × 10^−5^; 2.39 × 10^−8^	ZFP64, LINC01524, TSHZ2
20: 52,990,000–53,889,999	gain (3)	4.21 × 10^−24^; 0.00	TSHZ2, ZNF217, SUMO1P1
20: 53,890,000–61,649,999	gain (3)	7.00 × 10^−3^; 1.17 × 10^−3^	ANKRD60, APCDD1L, APCDD1L-DT, ATP5F1E, AURKA, BCAS1, BMP7, BMP7-AS1, C20orf85, CASS4, CBLN4, CDH26, CDH4, CSTF1, CTCFL, CTSZ, CYP24A1, DOK5, EDN3, FAM209A, FAM209B, FAM210B, FAM217B, GCNT7, GNAS, GNAS-AS1, LINC01440, LINC01441, LINC01711, LINC01716, LINC01718, LINC01742, LINC02910, MC3R, MIR296, MIR298, MIR4325, MIR4533, MIR4756, MIR548AG2, MIR646, MIR646HG, MTRNR2L3, NELFCD, NKILA, NPEPL1, PCK1, PFDN4, PHACTR3, PHACTR3-AS1, PMEPA1, PPP1R3D, PPP4R1L, PRELID3B, RAB22A, RAE1, RBM38, RBM38-AS1, RTF2, SLMO2-ATP5E, SPO11, STX16, SYCP2, TFAP2C, TUBB1, VAPB, ZBP1, ZNF831
20: 61,650,000–63,469,999	gain (3)	6.32 × 10^−37^; 0.00	ADRM1, ARFGAP1, BHLHE23, BIRC7, CABLES2, CDH4, CHRNA4, COL20A1, COL9A3, DIDO1, GATA5, GID8, HAR1A, HAR1B, HRH3, KCNQ2, KCNQ2-AS1, LAMA5, LAMA5-AS1, LINC00029, LINC00659, LINC01056, LINC01749, LSM14B, MIR1-1, MIR1-1HG, MIR1-1HG-AS1, MIR124-3, MIR1257, MIR133A2, MIR3195, MIR3196, MIR4326, MIR4758, MRGBP, MTG2, NKAIN4, NTSR1, OGFR, OGFR-AS1, OSBPL2, PSMA7, RBBP8NL, RPS21, SLC17A9, SLCO4A1, SLCO4A1-AS1, SNORA117, SS18L1, TAF4, TCFL5, WI2-87327B8.2, YTHDF1
20: 63,470,000–64,444,167	gain (3)	2.66 × 10^−4^; 3.03 × 10^−4^	ABHD16B, ARFRP1, C20orf181, C20orf204, DNAJC5, EEF1A2, FNDC11, GMEB2, HELZ2, KCNQ2, LIME1, LINC00266-1, LKAAEAR1, MHENCR, MIR1914, MIR647, MIR6813, MIR941-1, MIR941-2, MIR941-3, MIR941-4, MIR941-5, MYT1, NPBWR2, OPRL1, PCMTD2, PPDPF, PRPF6, PTK6, RGS19, RTEL1, SAMD10, SLC2A4RG, SOX18, SRMS, STMN3, TCEA2, TNFRSF6B, TPD52L2, UCKL1, UCKL1-AS1, ZBTB46, ZBTB46-AS1, ZGPAT, ZNF512B
21: 10,170,000–10,319,999	gain (3)	2.41 × 10^−3^; 7.19 × 10^−4^	none
X: 1–229,999	gain (7)	6.45 × 10^−6^; 2.95 × 10^−6^	none
X: 1,800,000–1,979,999	gain (3)	3.95 × 10^−4^; 8.63 × 10^−5^	none

## Data Availability

The raw sequence reads produced by this research were deposited in the NCBI Sequence Read Archive under BioProject PRJNA853293.

## Supplementary Materials

The following supporting information can be downloaded at: https://www.mdpi.com/article/10.3390/biomedicines10102588/s1, Figure S1: Western blots for proteins which resulted to be not expressed; Figure S2: Verification of the functionality of some antibodies. Figure S3: full data about Figure 6. Figure S4: magnification of Figure 6. Table S1: Literature analysis of genes identified by WGS-CNV analysis.
