# Supplementary material for: Effects of the Exposure of Human Non-Tumour Cells to Sera of Pancreatic Cancer Patients
Source: Biomedicines. 2022 Oct 15;10(10):2588. doi: 10.3390/biomedicines10102588 (PMC9599555; doi:10.3390/biomedicines10102588)
Supplement: Supplementary file 1 [file biomedicines-10-02588-s001.zip › biomedicines-1870956-supplementary.pdf]

TITLE:

Effects of the exposure of human non-tumour cells to sera of pancreatic cancer patients.

AUTHORS:

Berina Sabanovic, Matteo Giulietti, Monia Cecati, Gaya Spolverato, Clara Benna, Salvatore Pucciarelli, Francesco Piva

SUPPLEMENTARY MATERIAL

Supplementary Figure S1. Western blots for proteins which resulted to be not expressed. Western blots for E-cadherin (120 kDa), N-cadherin (100kDa), Fibronectin (220kDa). Lanes correspond to: (M) marker; (1) hTERT-HPNE + healthy serum; (2) hTERT-HPNE + PDAC serum; (3) HEK293T + healthy serum; and (4) HEK293T + PDAC serum.

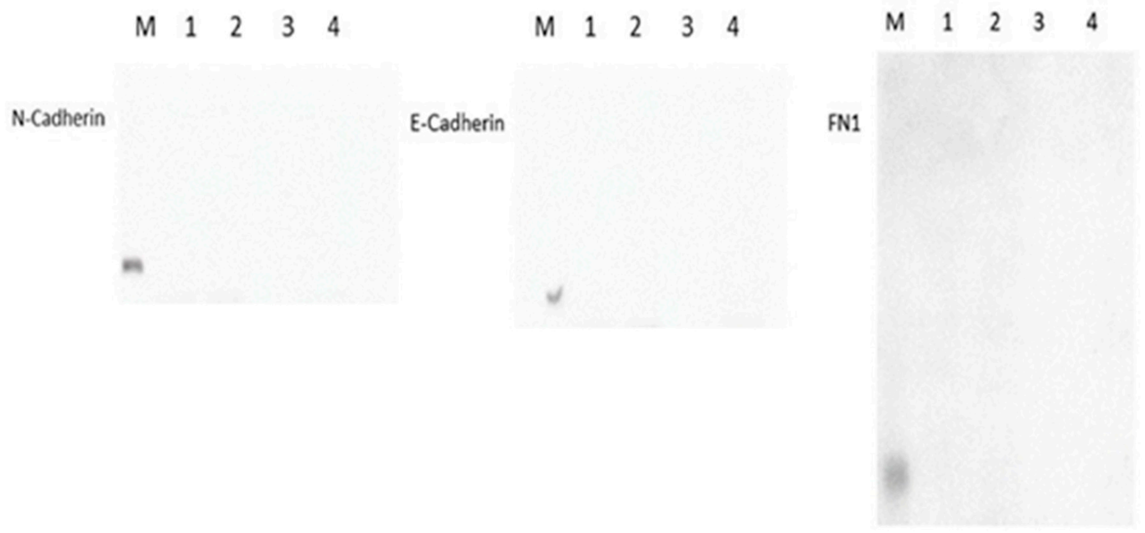

Supplementary Figure S2. Verification of the functionality of some antibodies. Western blots for Fibronectin (A), E-cadherin and N-cadherin (B). Cells have not been treated with human serum.

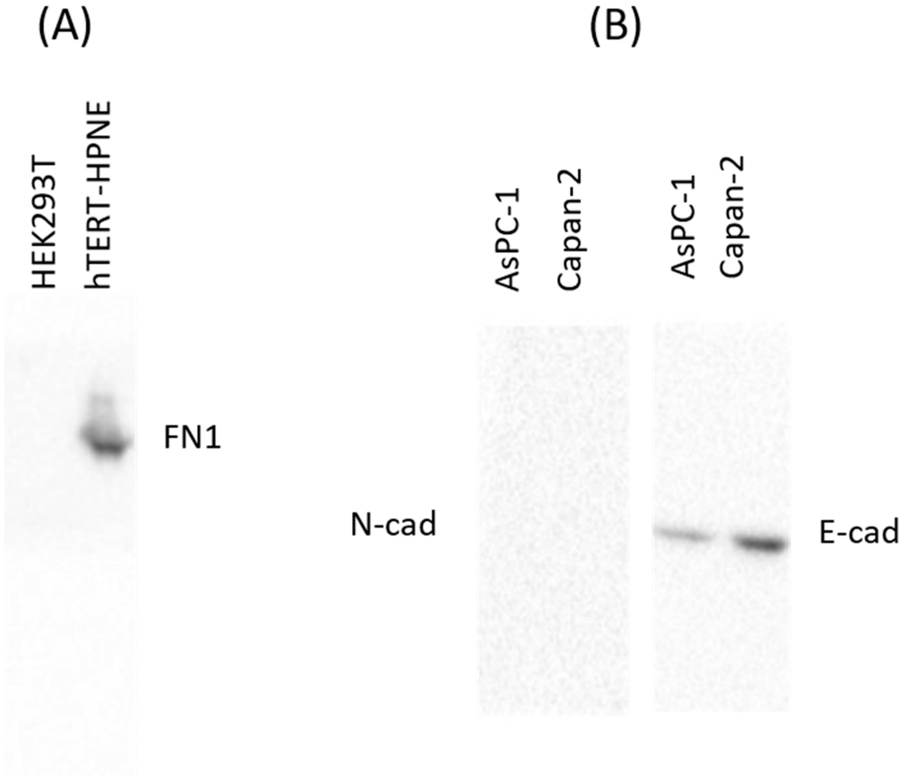

Supplementary Figure S3. These graphs summarize the results of the chromosomal gain or loss events of HEK293T cells, treated with plasma from patients, compared to cells treated with plasma from healthy subjects. An image is shown for each chromosome. The chromosomal position is shown on the abscissa axis. The number of copies of each specific chromosomal segment is shown in the ordinate axis. In the absence of CNV events, values equal to 2 (diploidy) are expected. Unfortunately, the size of this image does not allow for a good appreciation of the CNV events, therefore we have included the high-magnification image (Supplementary Figure S4) and more details are shown in Table 2.

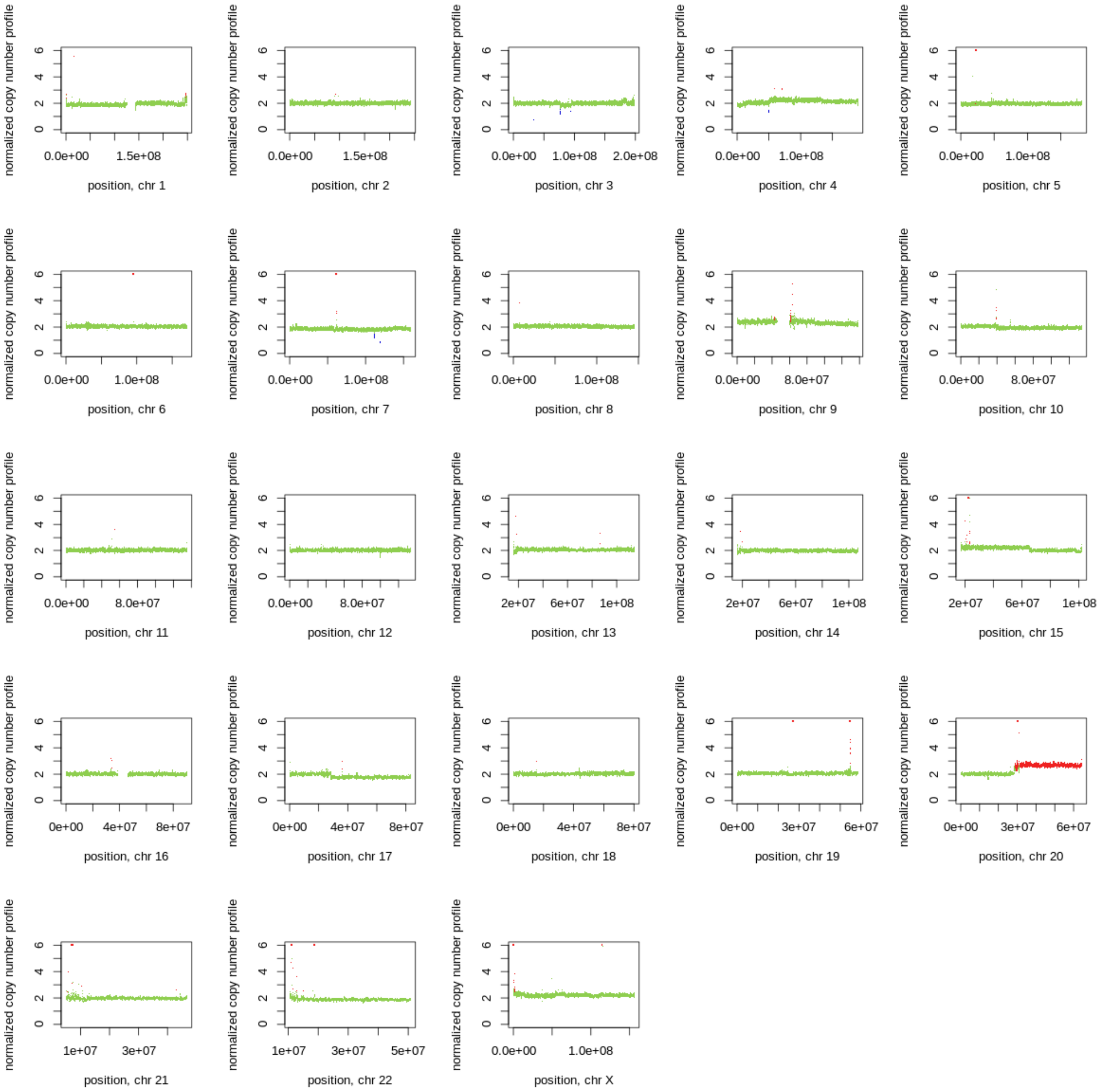

Supplementary Table S1. Literature analysis of genes identified by WGS-CNV analysis. Only cancer-associated literature is reported.

Lost Genes	Literature
FBXL2 (lost)	It has potential tumour suppressive functions by facilitating the degradation of some cell cycle regulators (cyclin D2, cyclin D3 and Aurora B) and, therefore, it induces cell cycle arrest (PMID: 26432751)
IMMP2L (lost)	It can suppress cell senescence (PMID: 29808012)
ROBO2 (lost)	In PDAC patients, ROBO2 expression is low and it is associated with poor survival, while its upregulation inhibited PDAC cell proliferation, migration, and invasion (PMID: 30504844, 34148491). It can be considered a tumor suppressor gene (PMID: 31868331).
**Gained Genes**	**Literature**
ADRM1	It promoted gastric epithelial cell proliferation by cell cycle progression, thus, is a driver of metastasis (PMID: 24968865)
ARFGAP1	ArfGAP1 has been identified as a key controller of mTORC1 complex in the regulation of cell growth by inhibiting mTOR activity. ArfGAP1 represses cell growth through mTORC1 and is an independent prognostic factor for the overall survival of pancreatic cancer patients (PMID: 33988249).
ATP5F1E	Down-regulation of a ATP synthase gene (ATP5F1A) is linked to the resistance to many anticancer drugs in various cancers, as for example the 5-FU resistance of colorectal cancer cells (PMID: 15833846). We have found another member of the ATP synthase complex, i.e., ATP5F1E to be amplified, together with significant sensitivity of treated cells to 5-FU.
AURKA	It plays crucial roles in mitotic spindle formation and centrosome maturation, and it is one of the fundamental tumour-linked genes also in PDAC (PMID: 15805292, PMID:33491761). Previously it has been reported that inhibition of AURKA expression using specific siRNA greatly reduced in vitro colony formation of various pancreatic cancer cell lines and improved their sensitivity to paclitaxel (PMID: 15805292). Remarkably, we have observed in our study that HEK293T cell line treated with PDAC patients' sera became resistant to paclitaxel, and a copy number variation of AURKA might be one of the causal factors.
BIRC7	BIRC7 has recently been identified as one of the key apoptosis regulators in PDAC and was correlated with poor prognosis. BIRC7 is overexpressed in many solid tumours, such as hepatocellular, gastric and breast cancer. Its role was linked with prognosis, tumour differentiation grade and invasiveness (PMID: 27802195). In PDAC tumour tissues, overexpression of BIRC7 has been demonstrated previously. Furthermore, it was reported that patients with higher BIRC7 expression survived much shorter than patients who did not express BIRC7. A significant correlation has been also observed among BIRC7 expression and tumour size, invasion and metastasis (PMID: 27802195).
BMP7	BMP7 is pleiotropic signalling molecule, a member of TGF-β protein superfamily, well-known for its role in cell development, growth, and invasion by altering target gene transcription. It was associated with metastasis and poor prognosis in many human tumours, and recently also with resistance to immunotherapy (PMID: 32973129, PMID: 27528760, PMID: 19056927). In PDAC, it was reported that BMP is important for initiation of EMT in a number of pancreatic cancer cell lines. Several BMP family members, including BMP7, enhance Slug expression, inhibit TbRIII expression and so promote EMT-associated invasion (PMID: 19056927).

CDH12	It is expressed in many cell types and plays important roles also in cancer development. Previous studies on non-small-cell lung cancer reported its role in cancer progression and correlation with poor prognosis (PMID: 19473719). Furthermore, its role has been demonstrated also in salivary adenoid cystic carcinoma, and recently colorectal cancer. It was reported that CDH12 is one of the key regulators of colorectal cancer invasiveness and metastasis by promoting EMT through activation of transcriptional factor Snail (PMID: 26762412, PMID: 24237488).
CDH4	Knockdown of CDH4 significantly desensitized pancreatic cancer cells to gemcitabine cytotoxicity (PMID: 27749787). Our results are in agreement, in fact the HEK293 cells treated with plasma from PDAC patients, where we observed an amplification of the CDH4 gene, are more sensitive to gemcitabine.
CTSZ	It is a tumorigenic protease able to promote tumour cell proliferation by interacting with integrins (PMID: 35444189)
CYP24A1	Paclitaxel biotransformation in the cell is mediated by members of cytochrome P450 protein family, which are usually found to be overexpressed or mutated in the paclitaxel-resistant clones (PMID: 15828850, PMID: 16550168). Here, we have found that CYP24A1, a gene for cytochrome P450 subunit, was amplified in PDAC serum treated cells.
DNAJC5	In hepatocellular carcinoma, it promotes cell proliferation (PMID: 33662413)
EDN3	It is a essential mitogen factor highly produced by glioblastoma stem cells in order to maintain stem cell migration, undifferentiation, and survival (PMID: 22013079)
EEF1A2	EEF1A2 is highly expressed in PDAC, its expression is associated with lymph node metastasis and with poor prognosis (PMID: 23165190). In PDAC, it promotes cell migration, invasion and metastasis (PMID: 23739844)
GATA5	In prostate cancer, GATA5 acts as a tumour suppressor (PMID: 35565203). Overexpression of GATA5 significantly inhibits the progression of colorectal cancer cells (PMID: 19509152)
GNAS	GNAS is considered a PDAC driver gene, i.e., a gene whose mutations cause tumour growth (PMID: 29941929).
GNAS-AS1	In nasopharyngeal carcinoma, this long non-coding RNA promotes cell migration and invasion via regulating Wnt/β-catenin pathway (PMID: 32271425)
HAR1A	It has a possible tumour suppressor role, since its upregulation increased the survival rates of patients who underwent radiotherapy and chemotherapy (PMID: 29108264). Moreover, mesenchymal markers N-cadherin, fibronectin, vimentin and slug were upregulated after HAR1A silencing (PMID: 34097087)
HAR1B	It is a predictive biomarker in bone and soft-tissue sarcoma cell lines (PMID: 33907565). In in clear cell renal cell carcinoma, it was upregulated and correlated with poor overall survival (PMID: 32368310)
HRH3	Numerous studies have suggested that histamine plays a role as an autocrine or paracrine growth factor by binding to four different G protein-coupled receptors (histamine receptors H1, H2, H3, and H4), triggering various cell signalling pathways. The presence of different histamine receptors has been confirmed in various cancer types, such as colorectal, gastric, and hepatocellular (PMID: 33159174). The study on pancreatic cancer cell line PANC-1 suggested the important role of all 4 types of histamine receptors in the promotion of tumour growth. In particular, histamine receptor 3 has been linked to the cell cycle regulation and tumour proliferation (PMID: 18345506).
KIR2DL1, KIR2DL4, KIR2DS4, KIR3DL1,	Killer cell immunoglobulin-like receptor genes (KIRs) that have been studied in solid tumours and correlated with immune evasion, however their role in PDAC is still unknown (PMID: 21342183, PMID: 34244312).

KIR3DL2, KIR3DL3, KIR3DL4	
LAMA5	It is a tumour-promoting protein upregulated in PDAC extracellular vesicles (PMID: 35241737). Synthesis and deposition of Laminin 5 (LAMA5) by carcinomas has been proposed as a novel mechanism of their invasion. Previous studies have offered new proofs for the role of basal membrane and function of different laminin-integrin receptors for cancer cell mobility. This feature was also studied in the PDAC cell lines, confirming that PDAC cells produce high quantities of laminin-5 and deposit it in the basement membrane enabling the cell migration using alpha 3 beta 1 integrin receptor recognizing laminin-5 (PMID: 9358755).
LINC00266-1	It stimulates the proliferative and metastatic abilities, and suppressed the apoptosis of osteosarcoma cells (PMID: 32709857)
LINC00659	It acts as an oncogene in colorectal cancer (PMID: 29523145, 33407563) and it is associated with poor prognosis in gastric cancer (PMID: 34084224).
LINC01711	It promotes metastasis in esophageal squamous cell carcinoma (PMID: 34370713)
LSM14B	Its overexpression in hepatocellular carcinoma correlated with poor clinical outcomes, i.e. higher TNM stage, advanced histologic grade, and worse prognosis (PMID: 35646684)
MHENCR	This lncRNA is involved in melanoma progression by regulating PI3K-Akt pathway (PMID: 28123636)
MIR1-1	Mir-1 acts as a consistent tumour suppressor gene. Downregulation of miR-1 has been demonstrated in multiple cancers, including pancreatic cancer (PMID: 34034986)
MIR124-3	Downregulation of miR-124 is associated with poor prognosis in PDAC patients (PMID: 27922430). It inhibits PDAC growth by regulating lactate metabolism (PMID: 30355947). When delivered by exosomes, it suppresses cell proliferation, epithelial mesenchymal transition, and enhance 5-FU sensitivity of pancreatic cancer cells (PMID: 33040049). Our results are in agreement, in fact the HEK293 cells treated with plasma from PDAC patients, where we observed an amplification of the mir-124 gene, are more sensitive to 5-FU.
MIR133A2	MicroRNA-133a has been proven downregulated in many cancer types and correlated with tumour progression. In pancreatic cancer, miR-133a expression was downregulated and correlated with aggressive clinicopathological features and poor survival. The transfection of this tumor suppressor miRNA into PANC-1 cells reduced tumorigenicity, cell proliferation, invasion, and migration and promoted apoptosis (PMID: 25198665)
MIR296	miR-296 known for its role in PDAC as a tumour suppressor by targeting AKT2 (PMID: 28534950).
MIR298	miR-298 overexpression promoted proliferation and metastasis in colorectal cancer (PMID: 31621072). Conversely, miR-298 suppresses the malignant progression of osteosarcoma (PMID: 35442509)
MIR3195	miR-3195 was downregulated in non-small cell lung cancer, and correlated with lymph node metastasis and TNM stage (PMID: 35046724)
MIR3196	In gastric cancer, miR-3196 acts as a tumour suppressor and it can be used to predict survival outcomes (PMID: 32419651).
MIR4326	In lung cancer, miR-4326 promotes proliferation by silencing the tumour suppressor gene APC2 (PMID: 29101731)
MIR646	Knockdown of miR-646 repressed proliferation and invasion ability of pancreatic cancer, while its overexpression was correlated with advanced tumour stage, lymphatic invasion, metastasis and poor overall survival in PDAC patients (PMID: 29343850)

MIR647	miR-647 was downregulated in gastric cancer, and correlated with reduced tumour size and metastasis, indicating antitumorigenic effects (PMID: 28098914). It inhibits glioma cell proliferation and invasion (PMID: 31881106). It also inhibits hepatocellular carcinoma cell proliferation (PMID: 34969357). Conversely, miR-647 showed a tumour promoter role in colorectal cancer (PMID: 28990086) and in gastric cancer (PMID: 30106095)
MIR941-1, MIR941-2, MIR941-3, MIR941-4, MIR941-5	MiR-941 is significantly upregulated in serum exosomes of laryngeal squamous cell carcinoma patients and miR-941 overexpression promoted cell proliferation and invasion (PMID: 32742479). Mir-941 was significantly upregulated in breast MDA-MB-231 cells, and its inhibition prevents cell proliferation and improves the sensitivity to 5-fluorouracil (PMID: 33087811). Our results are not in agreement, in fact HEK293 cells treated with plasma from PDAC patients, where we observed an amplification of MIR-941, are more sensitive to 5-fluorouracil.
MRGBP	MRGBP binds to protein components of histone acetyltransferase and deacetyltransferase complexes, and its upregulation is related to cell proliferation, migration, and invasion in many cancer types, including PDAC. It has been reported that MRGBP knockdown inhibited proliferation and migration of PDAC cells, and its overexpression had inverse effects promoting EMT. It has been proposed as a novel biomarker for PDAC for diagnostic and therapeutic approaches (PMID: 28969065).
NELFCD	In colorectal cancer, its upregulation promotes cell proliferation, migration, and invasion (PMID: 30584332)
NKILA	This lncRNA inhibits the proliferation and promotes the apoptosis of cervical squamous cell carcinoma (PMID: 31950510), inhibits tumour metastasis by suppressing EMT in hepatocellular carcinoma (PMID: 32015685), reduces oral squamous cell carcinoma development (PMID: 33143574)
NTSR1	It is overexpressed in advanced PDAC and correlated with poor prognosis. It also promotes metastatic abilities (PMID: 33034134)
OGFR	OGFR represses cell growth by the cyclin-dependent kinase inhibitor (CKI) p16 and regulates transition from G1 to S phase of cell cycle. The action of OGF on the p21 pathway has been demonstrated in several pancreatic cell lines, namely PANC-1, Capan-2 and BxPC-3 (PMID: 18190706).
PCK1	Phosphoenolpyruvate carboxykinase 1 (PCK1) is overexpressed in pancreatic cancer. Its silencing inhibited cell growth, proliferation, migration and invasion (PMID: 34620839)
PFDN4	In colorectal cancer patients with higher PFDN4 expression had a better prognosis (PMID: 20552408). Conversely, in gastric cancer, high mRNA expression of PFDN4 was associated with poor overall survival (PMID: 31957800)
PMEPA1	It induces the cell proliferation in pancreatic cancer via activation of the MAPK signalling pathway (PMID: 33857498). In PDAC, PMEPA1 is overexpressed and associated with disease-free survival. Its downregulation inhibited PDAC proliferation, invasion and migration, and enhanced the sensitivity to gemcitabine and cisplatin. Our results are not in agreement, in fact HEK293 cells treated with plasma from PDAC patients, where we observed an amplification of PMEPA1, are more sensitive to gemcitabine.
PPDPF	Higher levels of pancreatic progenitor cell differentiation and proliferation factor (PPDPF) are associated with increased cancer progression and poorer prognosis in hepatocellular carcinoma (PMID: 30817571) and non-small cell lung cancer (PMID: 34975328)
PSMA7	This proteasome subunit is overexpressed and associated with liver metastasis in colorectal cancer (PMID: 18202793) and gastric cancer (PMID: 33721161) and predicts poor prognosis in patients with gastric cancer (PMID: 31612044). Conversely, PSMA7 inhibits the tumorigenicity of lung adenocarcinoma cells (PMID: 22584585)
PTK6	Its overexpression enhanced cellular migration and invasion in PDAC (PMID: 24788754). Moreover, PTK6 enhanced apoptosis induced by gemcitabine, indeed PTK6 gene silencing increased cell survival after gemcitabine treatment (PMID: 26013168). Our results are in agreement, in fact HEK293 cells treated with plasma from PDAC patients, where we observed an amplification of PTK6, are more sensitive to gemcitabine.
RAB22A	RAB22A is overexpressed in PDAC patients, and the higher expression is associated with poorer survival (PMID: 32759795)

RAE1	RAE1, a mitotic checkpoint regulator, was amplified and overexpressed in colorectal cancer. The higher expression is associated with metastases and poor survival. It promoted tumour growth, inhibited apoptosis and induced paclitaxel resistance (PMID: 34008277). Our results are in agreement, in fact HEK293 cells treated with plasma from PDAC patients, where we observed an amplification of RAE1, are more resistant to paclitaxel.
RBM38	This RNA-binding protein frequently plays a tumour-suppressive role in multiple human cancer types (PMID: 32642788)
RGS19	RGS19 inhibits Ras activation by upregulating the tumour metastasis suppressor Nm23 (PMID: 23416464)
RUFY3	RUFY3 promotes EMT and, so, metastasis in colorectal cancer (PMID: 28089833, PMID: 28623323), in lung adenocarcinoma (PMID: 31772661), in hepatocellular carcinoma (PMID: 34510031). It induces the formation of F-actin-enriched protrusive structures at the cell periphery involved in cell migration, contributing to the metastatic potential of gastric cancer cells (PMID: 25766321). This evidence is in agreement with our data, indeed, in wound healing assay we demonstrated that treated HEK293T gained a significant migration ability.
SALL4	The transcription factor SALL4 regulates a network of transcriptional factors involved in stemness, pluripotency and chromatin remodelling not only in normal stem cells but also in cancer cells. Indeed, SALL4 expression has been associated with several cancer types (PMID: 23363002).
SLC17A9	Its high expression is associated with poor prognosis in colorectal cancer (PMID: 30236596), in in gastric carcinoma (PMID: 31799885) and in hepatocellular carcinoma (PMID: 32934749)
SLCO4A1	It is overexpressed in PDAC (PMID: 23307416) and in colorectal cancer, where it is associated with poor prognosis (PMID: 28378090)
SLCO4A1-AS1	This lncRNA induced pancreatic cancer cell proliferation, migration, invasion, and inhibited apoptosis (PMID: 35233463)
SOX18	Its overexpression is associated with enhanced cell growth and poor prognosis in PDAC (PMID: 27663663)
STMN3	It was overexpressed in lung adenocarcinoma and squamous cell carcinoma tissues and induced tumor cell proliferation, migration, and matrix invasion (PMID: 19258502).
TFAP2C	Its overexpression resensitizes pancreatic cancer cells to gemcitabine (PMID: 29615098). We found TFAP2C to be amplified in our HEK293T PDAC serum treated cell line and we observed a significant sensitivity to gemcitabine treatments.
TNFRSF6B	It is overexpressed in PDAC and associated with poor prognosis. Its knockdown expression inhibited pancreatic cancer cell proliferation and invasion (PMID: 34532119, PMID: 31025496). In PDAC, it is frequently amplified and its higher expression correlated with staging, tumour size, lymph node metastasis and histological staging (PMID: 22524850)
TPD52L2	In lung adenocarcinoma, it can be considered as a prognostic biomarker (PMID: 34744715)
TSHZ2	It is lowly expressed in lung adenocarcinoma and its ectopic overexpression inhibited cell proliferation, colony formation ability, migration and apoptosis (PMID: 33850468)
TUBB1	It is overexpressed in urothelial carcinoma of the bladder and associated with poor recurrence-free survival (PMID: 23184177)
UCKL1	It was overexpressed in sera of breast cancer patients (PMID: 34967537) and prostate cancer (PMID: 35548962). It protects against natural killer (NK) cell killing activity (PMID: 35583288)
VAPB	Its aberrant expression is associated with breast cancer, and correlated negatively with patient survival (PMID: 23049696)
YTHDF1	It is known to be overexpressed in many solid tumours including PDAC (PMID: 34026603).
ZBTB46	In metastatic castration-resistant prostate cancer, it acts as a tumour promoter since its overexpression promotes AR-independent proliferation (PMID: 28692046)

ZFP64	In gastric cancer, its overexpression was associated with aggressive phenotypes and poor prognosis. It was also associated with paclitaxel resistance (PMID: 34996504). Our results are in agreement, in fact HEK293 cells treated with plasma from PDAC patients, where we observed an amplification of ZFP64, are more resistant to paclitaxel.
ZNF217	ZNF217 is defined as oncogene in many solid tumours, including PDAC, and its amplification and/or overexpression has been widely reported (PMID: 25824781, PMID: 22593193, PMID: 20661224, PMID: 35362545). ZNF217 is able to promote AURKA, also amplified in our HEK293T cells, and activates PI3K/Akt pathway (PMID: 19242095, PMID: 25824781). It promotes cancer cell invasion by impairing expression of E-cadherin which consequently induces EMT switch and gives rise to metastasis development (PMID: 25824781).

Supplementary Figure S4. Detailed images of Figure 6 of the analysis result of CNVs derived from NGS sequencing.

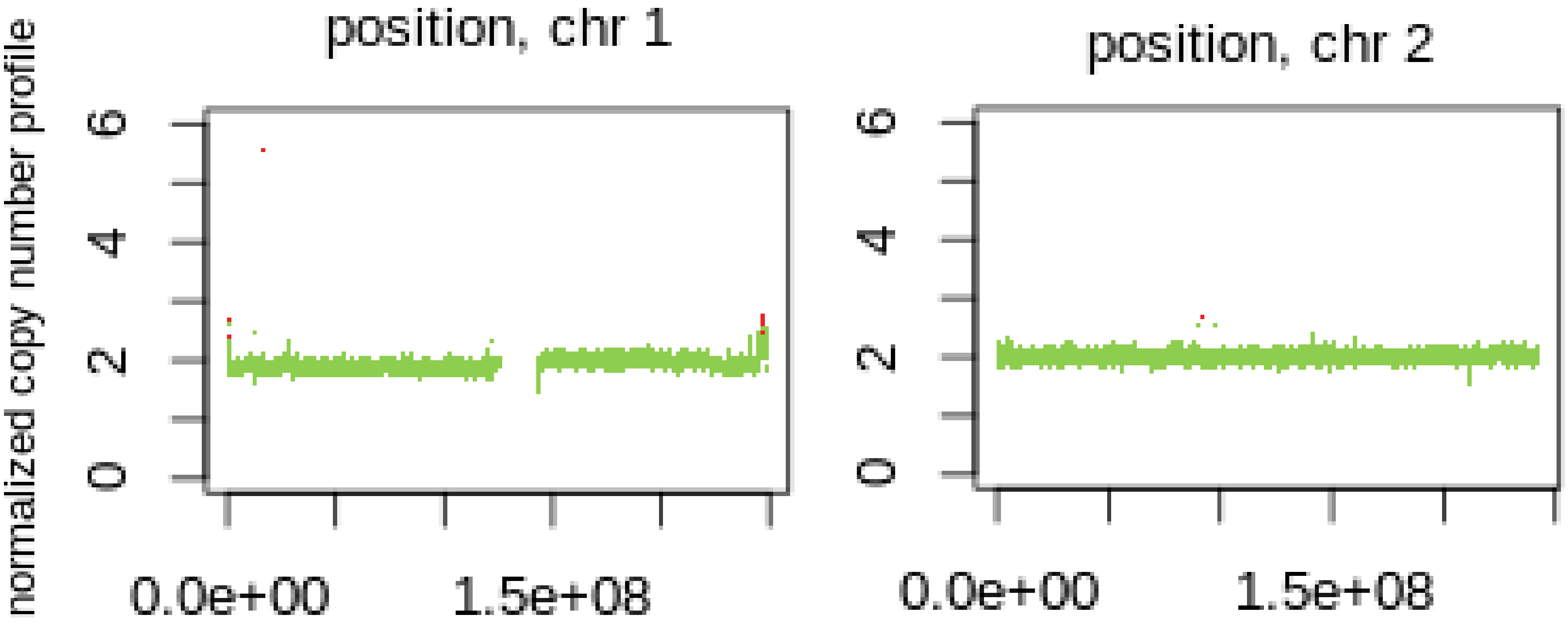

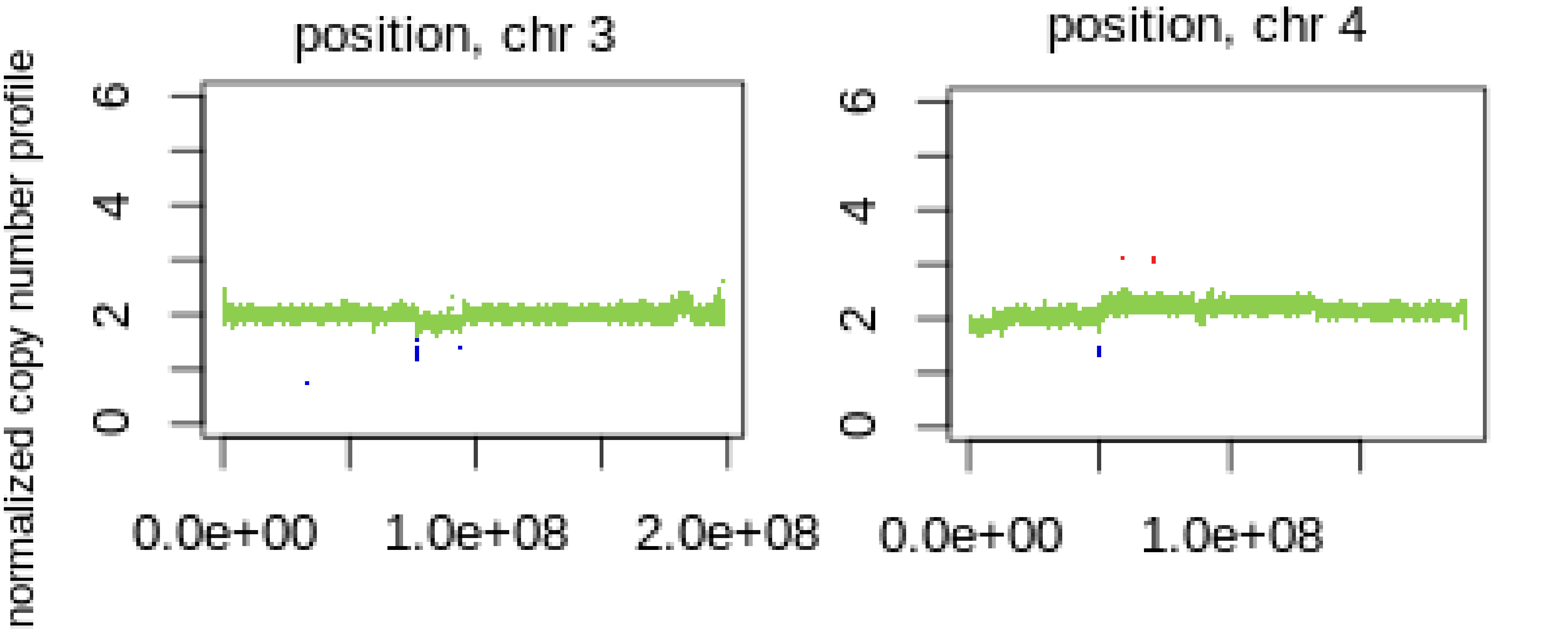
normalized copy number profile
position, chr 3
position, chr 4
0
2
4
6
0.0e+00
1.0e+08
2.0e+08

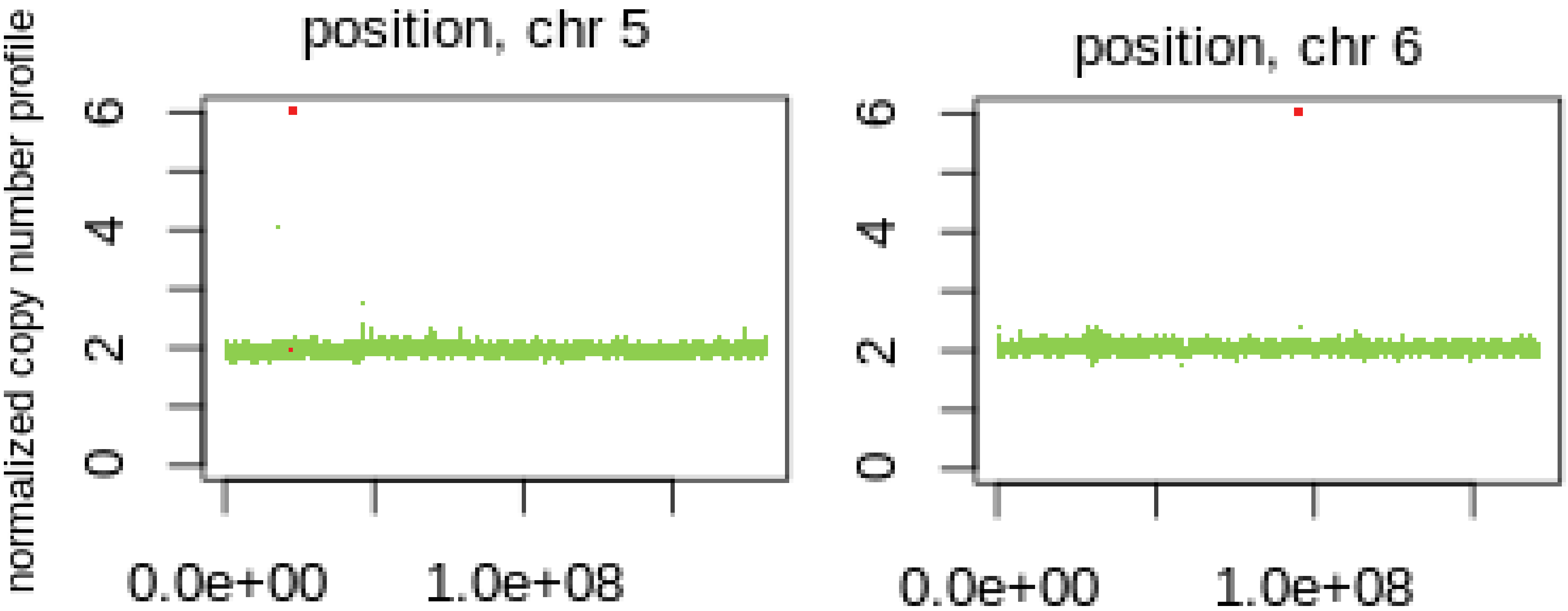
position, chr 5
position, chr 6
normalized copy number profile
0
2
4
6
0.0e+00
1.0e+08

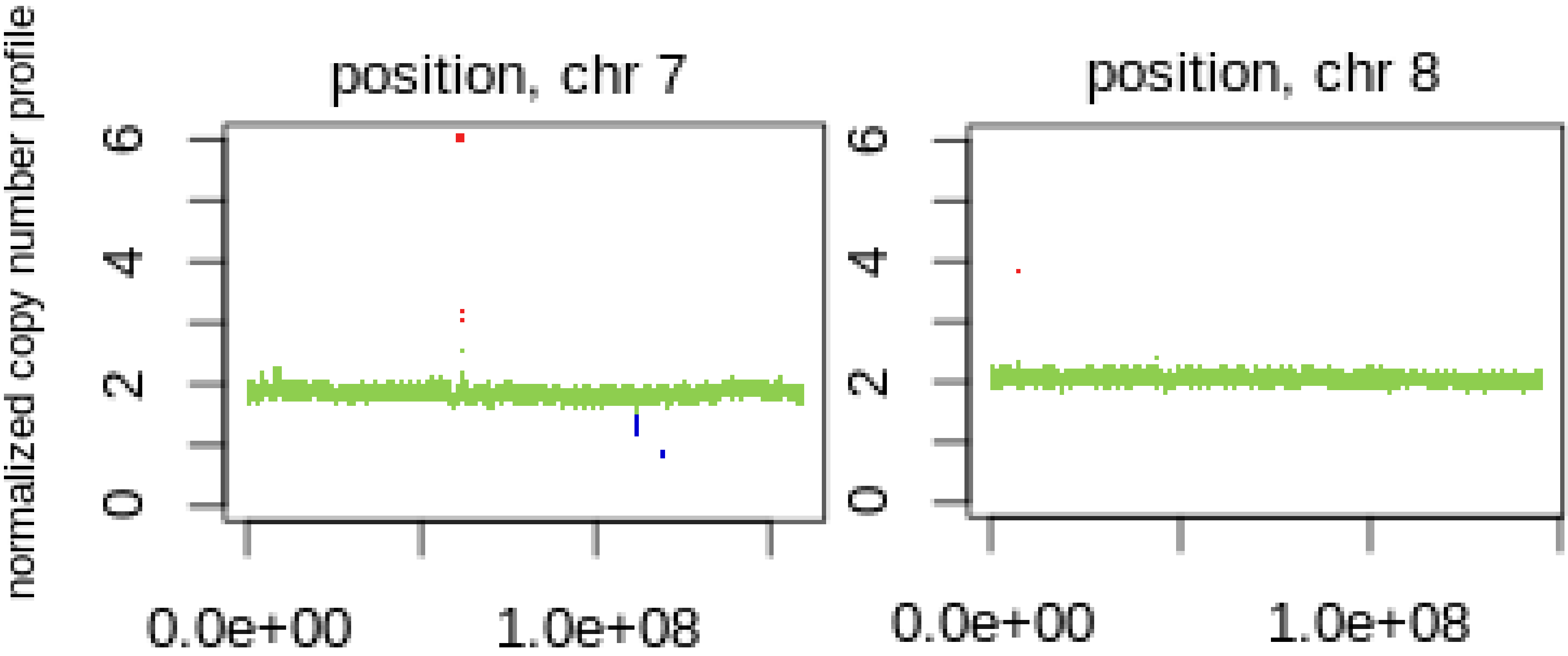

normalized copy number profile
position, chr 7
position, chr 8
0
2
4
6
0.0e+00
1.0e+08

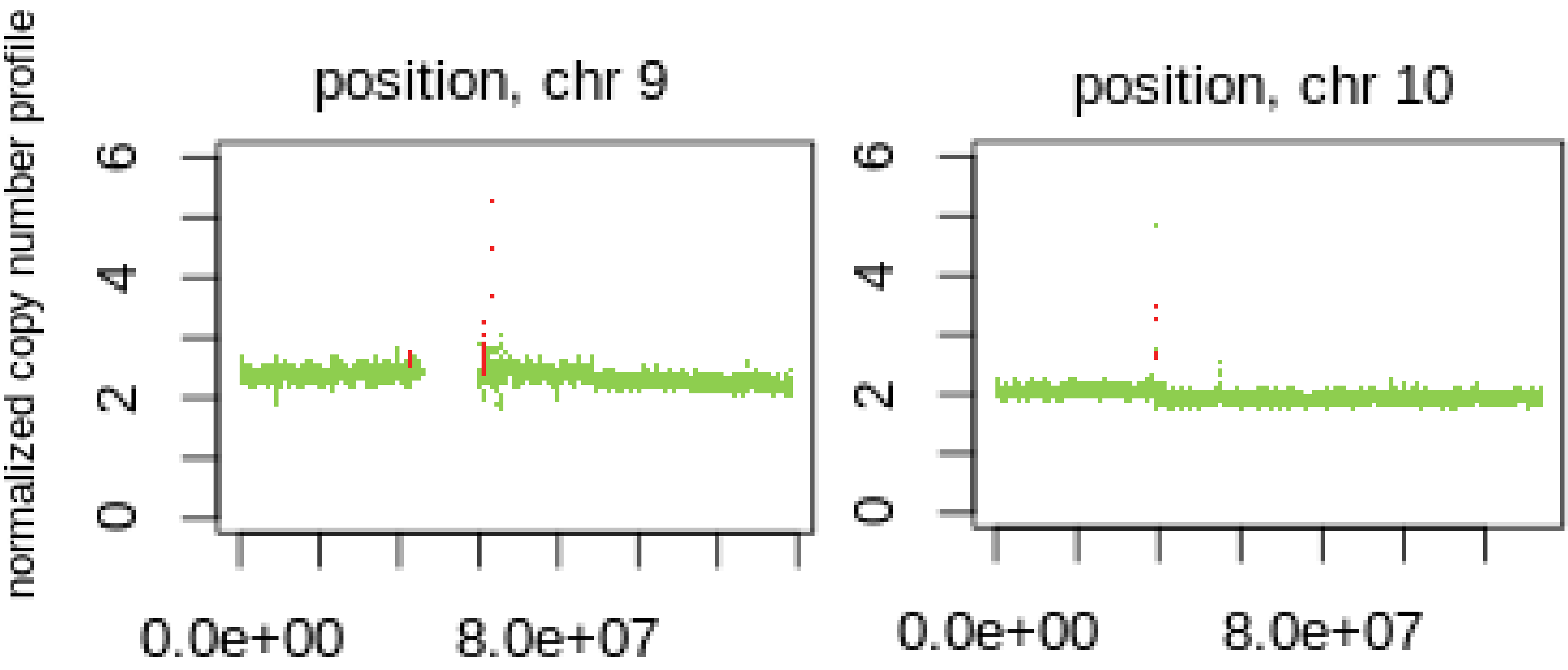
normalized copy number profile
position, chr 9
position, chr 10
0
2
4
6
0.0e+00
8.0e+07

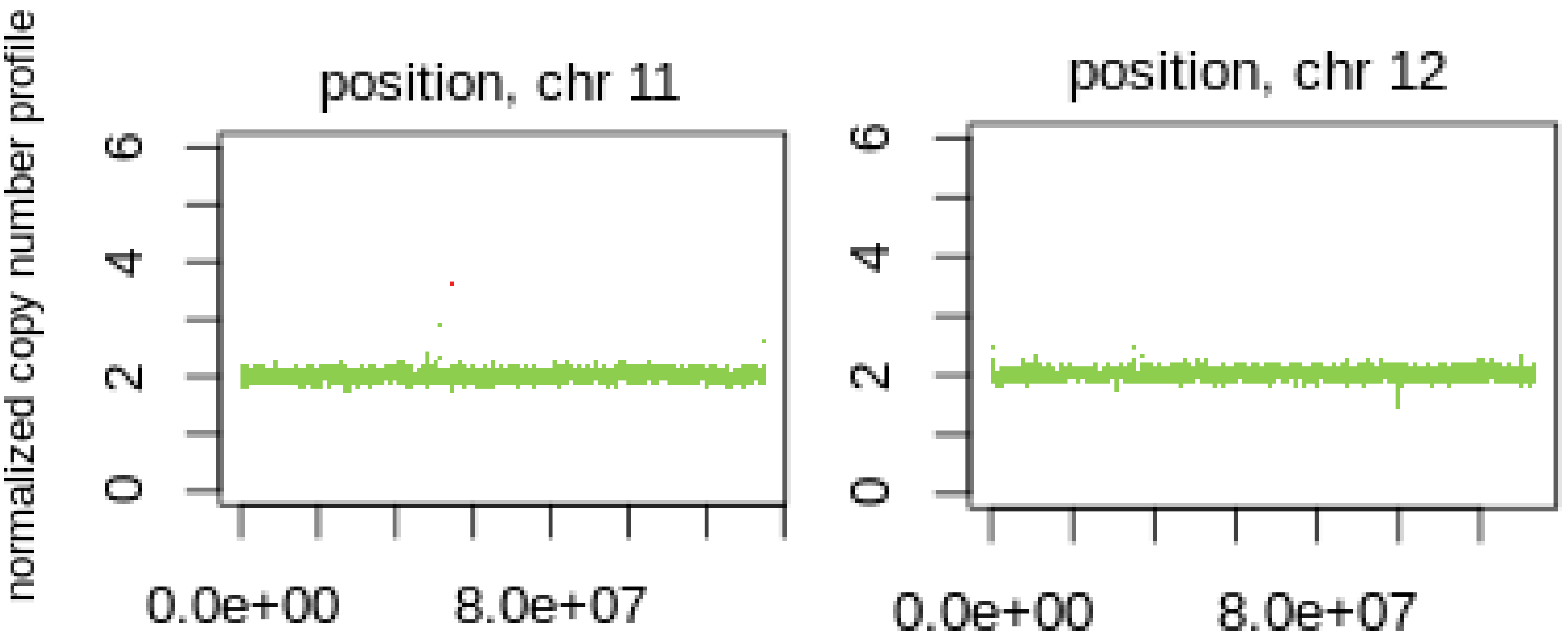

normalized copy number profile
position, chr 11
position, chr 12
0
2
4
6
0.0e+00
8.0e+07

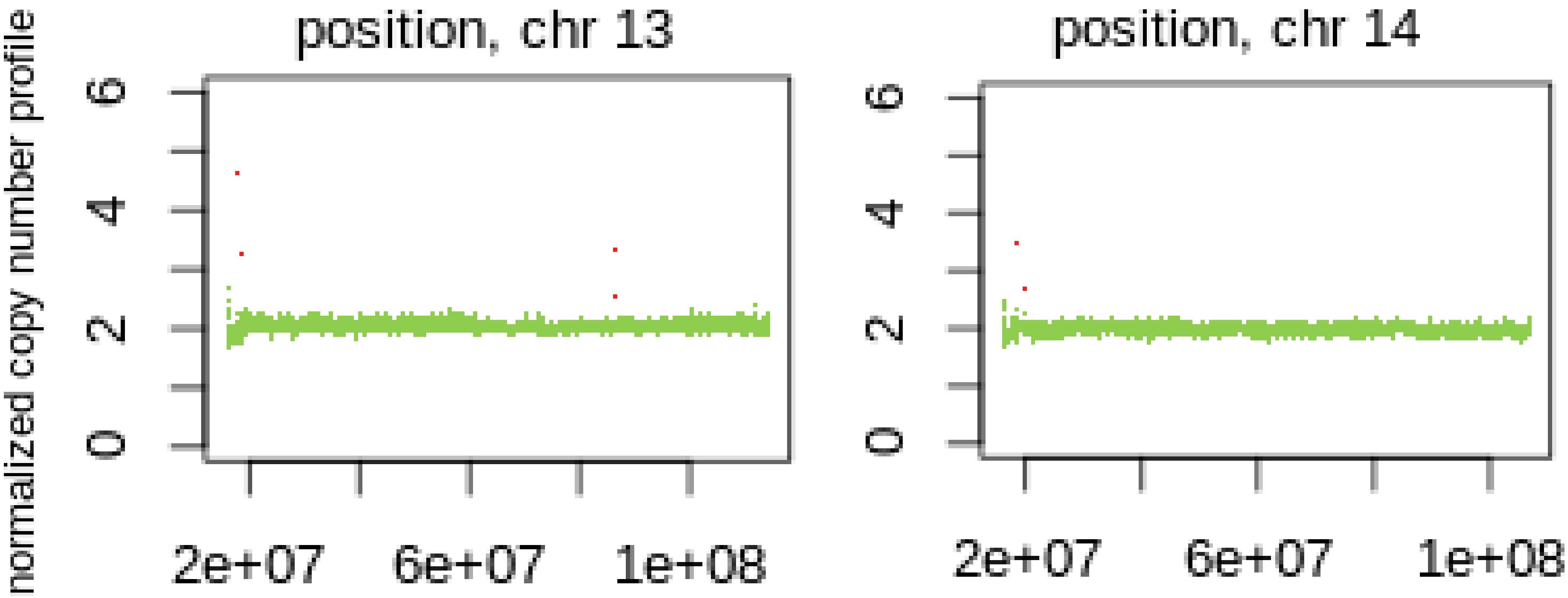
normalized copy number profile
position, chr 13
position, chr 14
0
2
4
6
2e+07
6e+07
1e+08

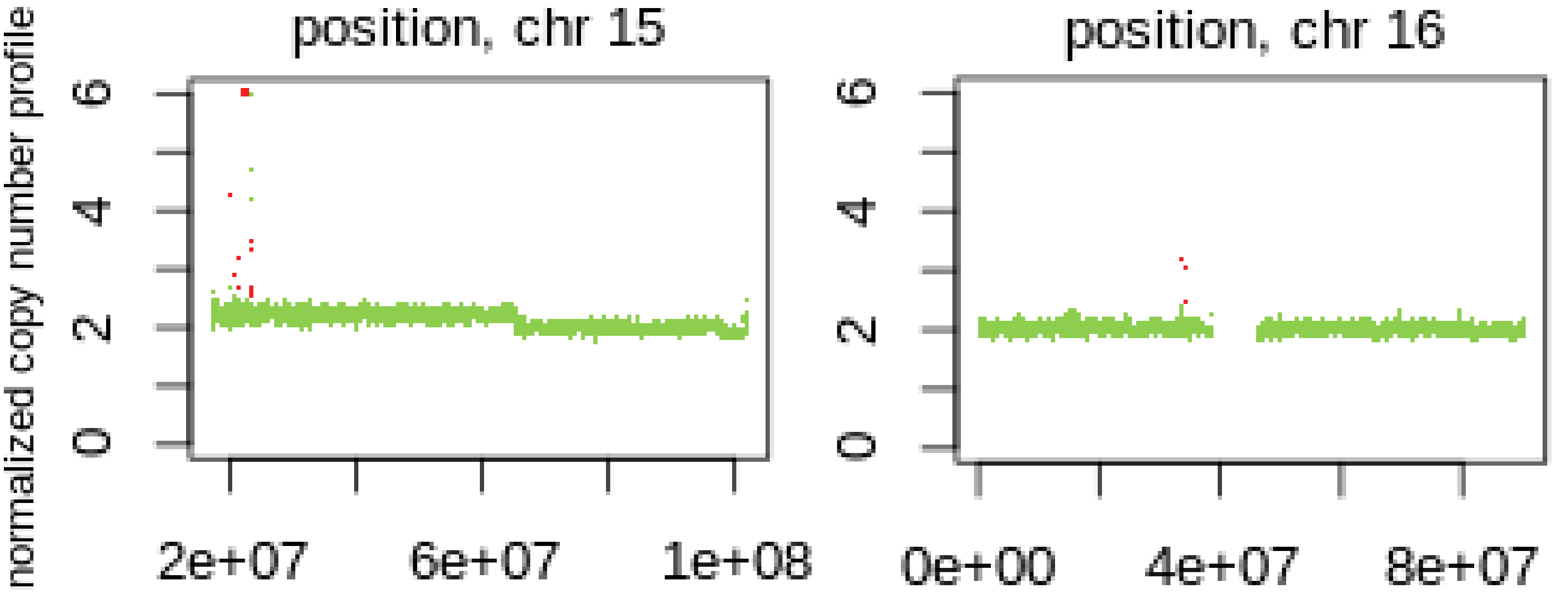
position, chr 15
position, chr 16
normalized copy number profile
0
2
4
6
2e+07
6e+07
1e+08
0e+00
4e+07
8e+07

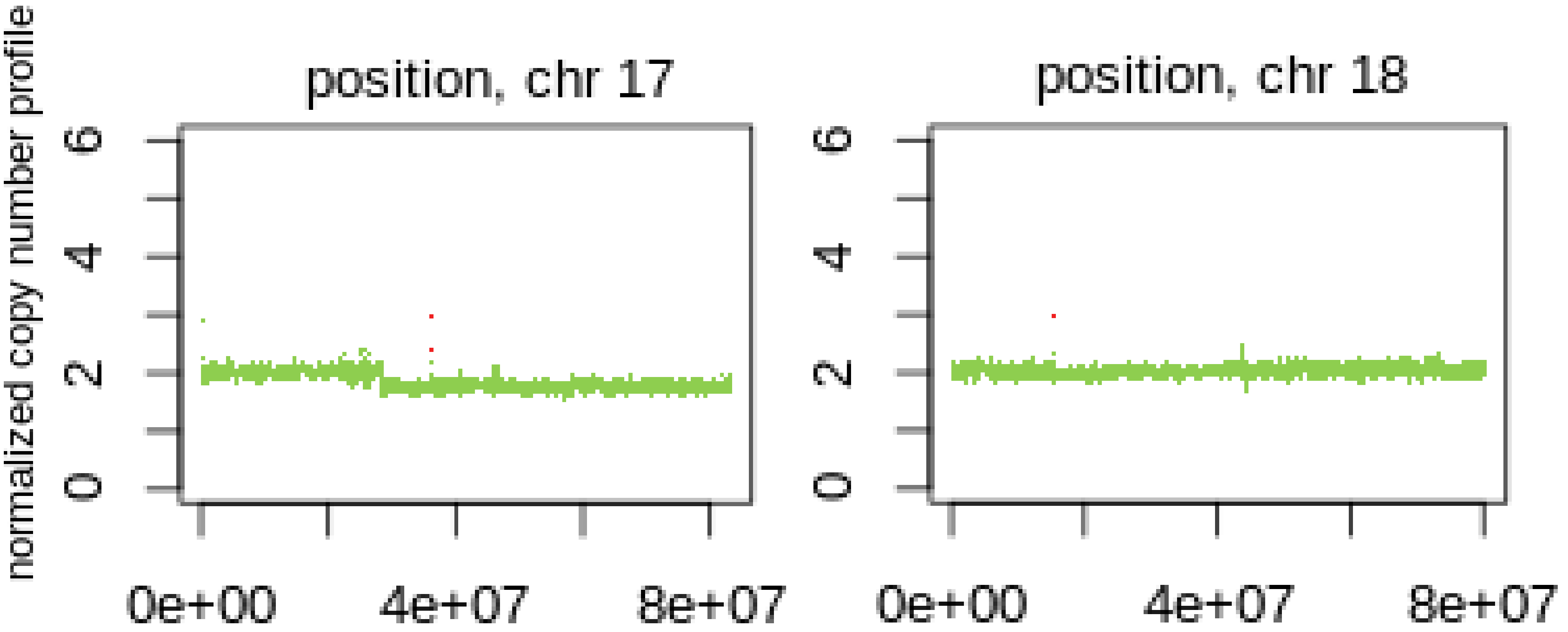
normalized copy number profile
position, chr 17
position, chr 18
0
2
4
6
0e+00
4e+07
8e+07

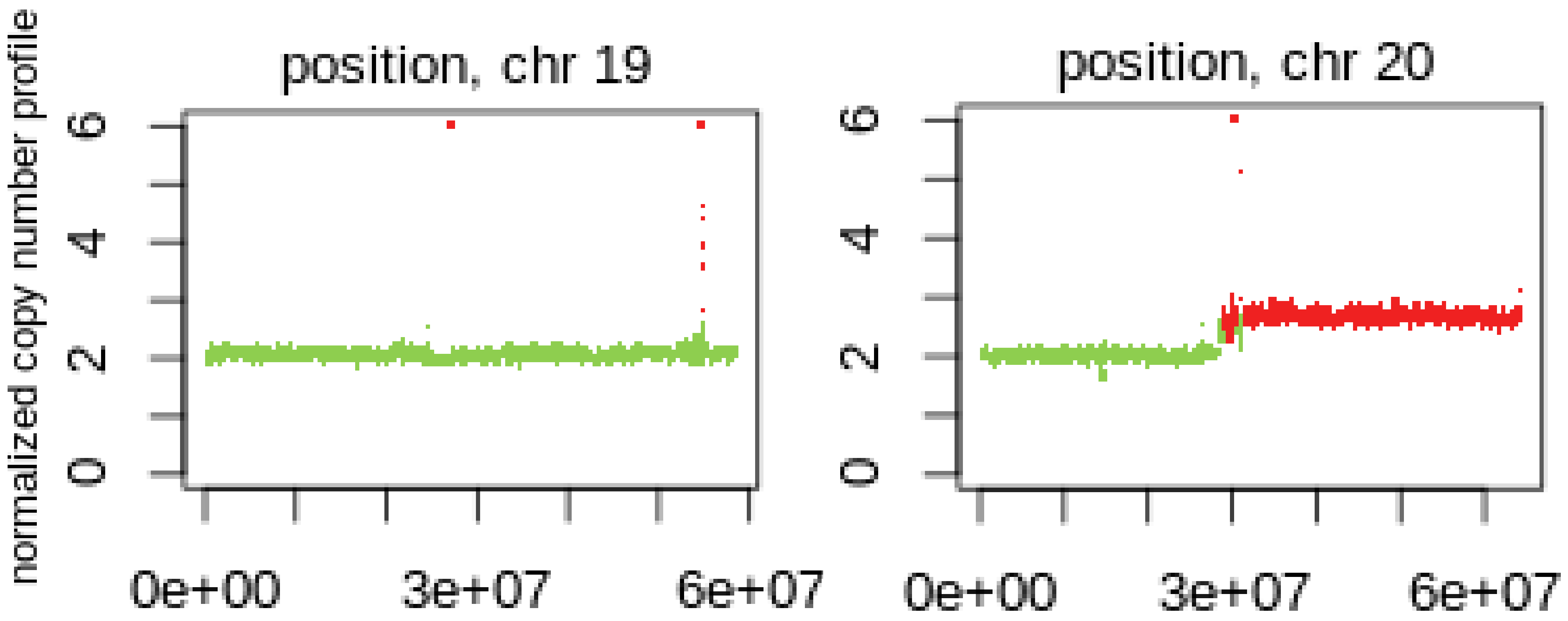

normalized copy number profile
position, chr 19
position, chr 20
0
2
4
6
0e+00
3e+07
6e+07

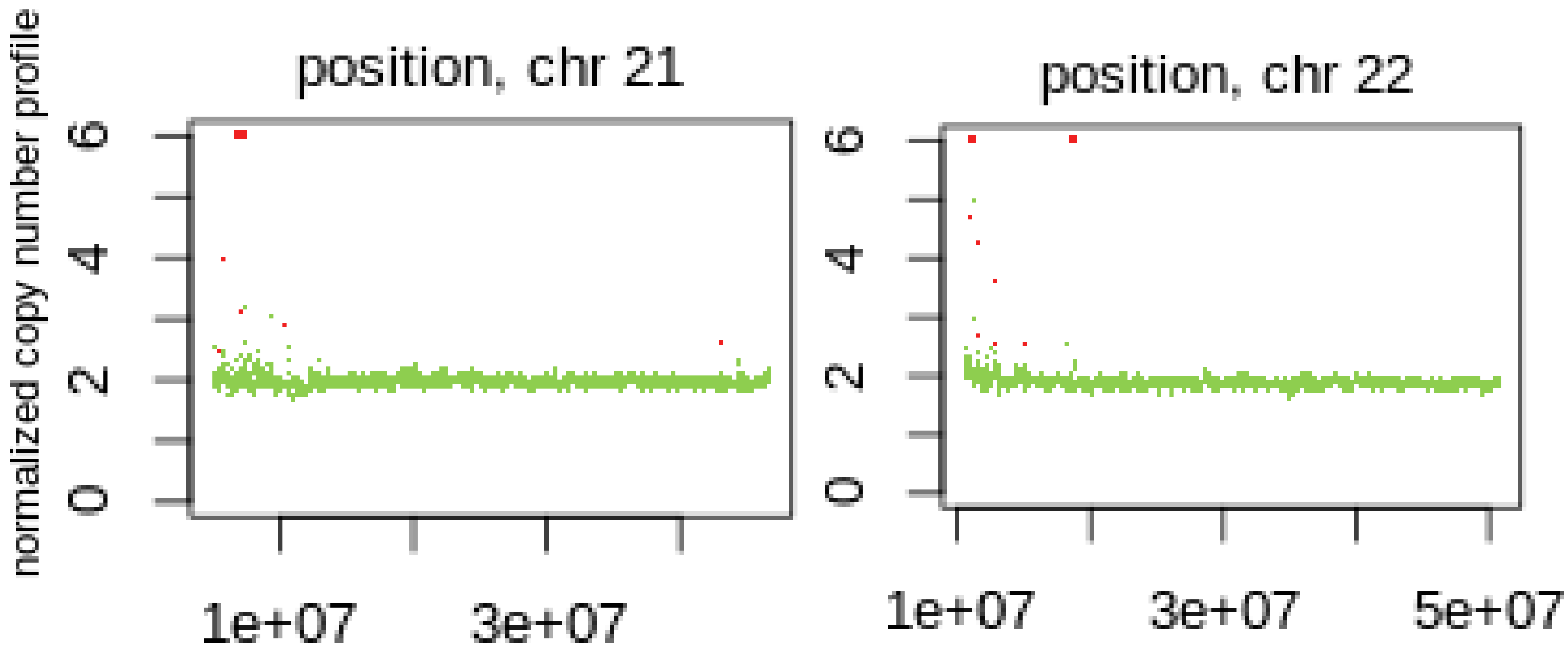
normalized copy number profile
position, chr 21
0
2
4
6
1e+07
3e+07
position, chr 22
0
2
4
6
1e+07
3e+07
5e+07

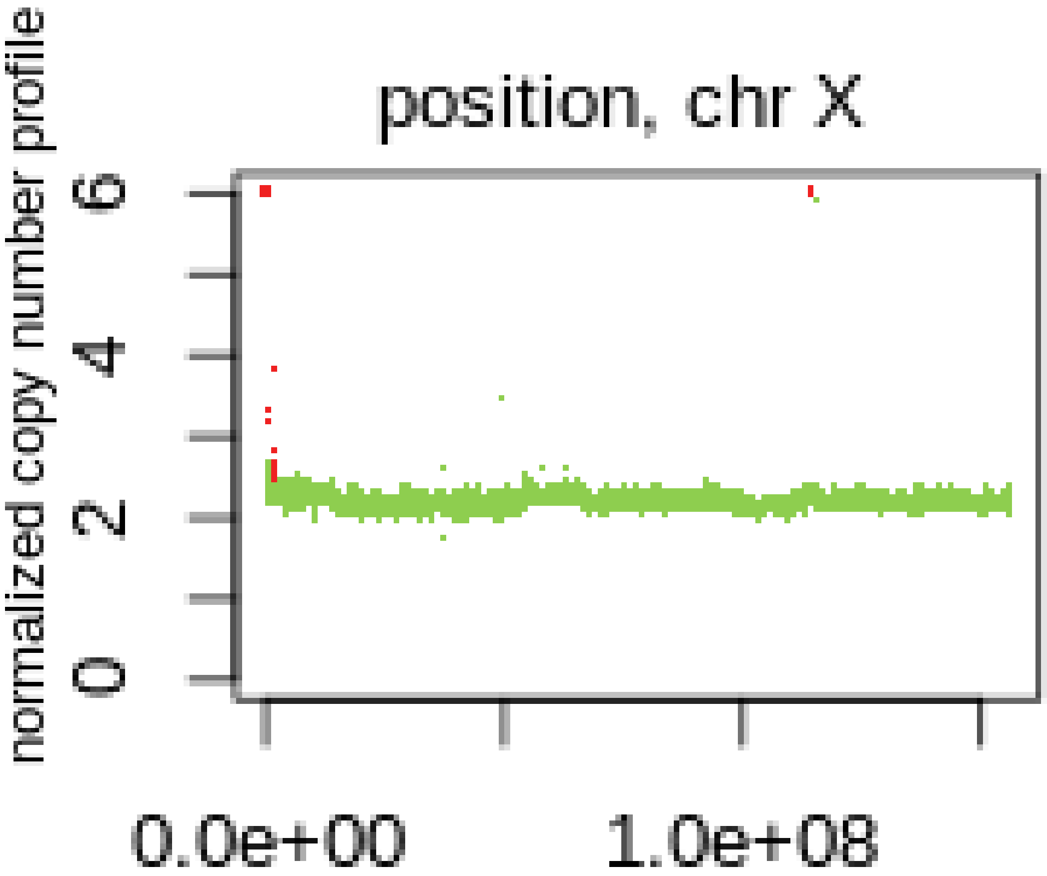
position, chr X
normalized copy number profile
0
2
4
6
0.0e+00
1.0e+08
